# Multi-Attribute Decision-Making Based on Bonferroni Mean Operators under Cubic Intuitionistic Fuzzy Set Environment

**DOI:** 10.3390/e20010065

**Published:** 2018-01-17

**Authors:** Gagandeep Kaur, Harish Garg

**Affiliations:** School of Mathematics, Thapar Institute of Engineering & Technology (Deemed University), Patiala, 147004 Punjab, India

**Keywords:** Bonferroni mean, aggregation operator, cubic intuitionistic fuzzy set, interval-valued intuitionistic fuzzy numbers, group decision-making

## Abstract

Cubic intuitionistic fuzzy (CIF) set is the hybrid set which can contain much more information to express an interval-valued intuitionistic fuzzy set and an intuitionistic fuzzy set simultaneously for handling the uncertainties in the data. Unfortunately, there has been no research on the aggregation operators on CIF sets so far. Since an aggregation operator is an important mathematical tool in decision-making problems, the present paper proposes some new Bonferroni mean and weighted Bonferroni mean averaging operators between the cubic intuitionistic fuzzy numbers for aggregating the different preferences of the decision-maker. Then, we develop a decision-making method based on the proposed operators under the cubic intuitionistic fuzzy environment and illustrated with a numerical example. Finally, a comparison analysis between the proposed and the existing approaches have been performed to illustrate the applicability and feasibility of the developed decision-making method.

## 1. Introduction

Decision-making is an important phenomenon to obtain the best-suited alternative among the available ones. In it, a number of researchers have presented a variety of concepts to reach the correct decisions. In primitive times, decisions were framed on the basis of crisp numbered data sets, but they were led to inadequate results having less applicability towards the real-life operational situations. However, with the passage of the time and due to the increase of the complexities in the system, it is difficult for the decision maker to handle the uncertainties in the data and hence the decisions under the traditional approach are unable to identify the best alternative. Thus, the researchers have represented the information in terms of fuzzy sets (FSs) [1], interval-valued fuzzy sets (IVFSs) [2], intuitionistic fuzzy sets (IFSs) [3], interval-valued intuitionistic fuzzy sets (IVIFSs) [4]. During the last decades, the researchers are paying more attention to these theories and have successfully applied it to the various situations in the decision-making process. Among these, an aggregation operator is an important part of the decision-making which usually takes the form of mathematical function to aggregate all the individual input data into a single one. For instance, Xu and Yager [5] developed some geometric aggregation operators to aggregate the different preferences of the decision-makers in the form of the intuitionistic fuzzy numbers (IFNs). Later on, Wang and Liu [6] extended these operators by using Einstein norm operations. Garg [7] had presented generalized intuitionistic fuzzy interactive geometric interaction operators using Einstein norm operations for aggregating the different intuitionistic fuzzy information. Garg [8], further, proposed some series of interactive aggregation operators for intuitionistic fuzzy numbers (IFNs). Garg [9] presented generalized intuitionistic fuzzy aggregation operators under the intuitionistic multiplicative preference relation instead of intuitionistic fuzzy preference relations. Garg [10] extended the theory of the IFSs to the Pythagorean fuzzy sets and presented their generalized averaging aggregation operators. Wang and Liu [11] presented some hybrid weighted aggregation operators using Einstein norm operators while Garg [12] presented some improved interactive aggregation operators. However, apart from that, some other authors have presented different methods such as ranking functions [13,14,15,16,17], aggregation operators [18,19,20,21,22,23,24,25] to solve the decision-making problems.

As the above aggregation operators have widely been used by the researchers during the decision-making (DM) process in which they have highlighted the importance of each factor or its ordered position but cannot reflect the interrelationships of the individual data. On the other hand, in our real-life situation, there always exists a situation in which a relationship between the different criteria such as prioritization, support, and impact each other plays a dominant role during an aggregation process. For handling it and to incorporate into the DM analysis, Yager [26] introduced the power average (PA) aggregation operator which allows argument values to support each other in the aggregation process. Further, Xu and Yager [27], Yu [28] investigated the prioritized averaging and geometric aggregation operators under IFS environment. Also, Yager [29] proposed the concept of the Bonferroni Mean (BM) [30] whose main characteristic is its capability to capture the interrelationship between the input arguments. Beliakov et al. [31] introduced the generalized Bonferroni mean to overcome the drawback of BM. Xu and Yager [32] developed an intuitionistic fuzzy Bonferroni mean to aggregate the intuitionistic fuzzy information. Xu and Chen [33] extended these mean operators to the IVIFSs environment. Xia et al. [34] proposed the generalized intuitionistic fuzzy BMs. Liu et al. [35] presented the partitioned BM operators under IFSs environment. Shi and He [36] threw light on optimizing BMs with their applications to various decision-making processes. Garg and Arora [37] presented BM aggregation operator under intuitionistic fuzzy soft set environment.

From the above existing literature, we can see that all the existing studies mainly focus on the fuzzy set, interval fuzzy set, IFS, IVIFS, and their corresponding applications. Later on, Jun et al. [38] introduced the concepts of the cubic sets (CSs) by the combination of both interval-valued fuzzy numbers and fuzzy number and defined some logic operations of the cubic sets. Under this set, Khan et al. [39] presented some cubic aggregation operators while Mahmood et al. [40] introduced the concepts of the cubic hesitant fuzzy sets and their aggregation operators in the decision-making process. However, above theories contain only the information in the form of membership intervals and do not stress on the non-membership portion of the data entities, which also play an equivalent role during assessing the alternative in the decision-making process. On the other hand, in the real world, it is often difficult to express the value of a membership function by an exact value in a fuzzy set. In such cases, it may be easier to describe vagueness and uncertainty in the real world using an interval value and an exact value, rather than unique interval/exact values. Thus, the hybrid form of an interval value and an exact value may be a very useful expression for a person to describe certainty and uncertainty due to his/her hesitant judgment in complex decision-making problems. For this purpose, we present the concept of the cubic intuitionistic fuzzy set (CIFS) which is described by two parts simultaneously, where one represents the membership degrees by an interval-valued intuitionistic fuzzy value and the other represents the membership degrees by intuitionistic fuzzy value. Hence, a CIFS is the hybrid set combined by both an IVIFN and an IFN. Obviously, the advantage of the CIFS is that it can contain much more information to express the IVIFN and IFN simultaneously. On the other hand, the CIFS contains much more information than the general intuitionistic set (IVIFS/IFS) because the CIFS is expressed by the combined information of both the sets. Hence, CIFS its rationality and effectiveness when used for evaluating the alternatives during the decision-making process since the general decision-making process may either use IVIFSs or IFSs information which may lose some useful evaluation information, either IVIFSs or IFSs, of alternatives, which may affect the decision results. Currently, since there is no study on aggregation operators which reflect the relationship between the different criteria of the decision-making process having cubic intuitionistic fuzzy information.

In the present communication, motivated by the concept of the Bonferroni mean and by taking the advantages of the CIFS to express the uncertainty, we propose some new aggregation operators called the cubic intuitionistic fuzzy Bonferroni mean (CIFBM), as well as weighted cubic intuitionistic fuzzy Bonferroni mean (WCIFBM) operator to aggregate the preferences of decision-makers. Various desirable properties of these operators have also been investigated in details. The major advantages of the proposed operator are that they have considered the interrelationships of aggregated values. Further, we examine the properties and develop some special cases of proposed work. Some of the existing studies have been deduced from the proposed operator which signifies that the proposed operators are more generalized than the others. Finally, a decision-making approach has been given for ranking the different alternatives based on the proposed operators.

The remainder of the article is organized as follows. Section 2 briefly describes some concepts of IFSs, IVIFSs, and CSs. Section 3 presents cubic intuitionistic fuzzy sets and the new aggregation operators called the cubic intuitionistic fuzzy Bonferroni mean (CIFBM) and weighted cubic intuitionistic fuzzy Bonferroni mean (WCIFBM) operators and discuss its particular cases. Some properties of these operators are also discussed here. In Section 4, a decision-making approach has been established, based on proposed operators, to solve the multi-attribute decision-making (MADM) problems. A numerical example is presented in Section 5 to illustrate the proposed approach and to demonstrate its practicality and effectiveness. The paper ends in Section 6 with concluding remarks.

## 2. Preliminaries

In this section, some basic concepts related to IFSs, IVIFSs etc., are reviewed briefly.

**Definition** **1.** Ref. [3] An intuitionistic fuzzy set (IFS) A defined over the universal set X is given as an ordered pair $A=\left\{(x,\zeta_{A}\left(x\right),\vartheta_{A}\left(x\right)),\forall x\in X\right\}$ where $0\leq \zeta_{A}\left(x\right)\leq 1$, $0\leq \vartheta_{A}\left(x\right)\leq 1$ and $\zeta_{A}\left(x\right)+\vartheta_{A}\left(x\right)\leq 1$. We denote this pair as $A=\langle \zeta_{A},\vartheta_{A}\rangle$ and name it as intuitionistic fuzzy number (IFN).

**Definition** **2.** *Let $A=\langle \zeta_{A},\vartheta_{A}\rangle$ and $B=\langle \zeta_{B},\vartheta_{B}\rangle$ be two IFNs. Then the following expressions are defined as [3]*

*(i)*  A⊆B if $\zeta_{A}\left(x\right)\leq \zeta_{B}\left(x\right)$ and $\vartheta_{A}\left(x\right)\geq \vartheta_{B}\left(x\right)$ for all x in X;
*(ii)*  A=B if and only if A⊆B and B⊆A.
*(iii)*  $A^{c}=\{x,\langle \vartheta_{A}\left(x\right),\zeta_{A}\left(x\right)\rangle |x\in X\rangle \}$
*(iv)*  $A\cap B=\{x,\langle \inf\left(\zeta_{A}\left(x\right),\zeta_{B}\left(x\right)\right),\sup\left(\vartheta_{A}\left(x\right),\vartheta_{B}\left(x\right)\right)\rangle |x\in X\}$
*(v)*  $A\cup B=\{x,\langle \sup\left(\zeta_{A}\left(x\right),\zeta_{B}\left(x\right)\right),\inf\left(\vartheta_{A}\left(x\right),\vartheta_{B}\left(x\right)\right)\rangle |x\in X\}$

After that, Atanassov and Gargov [4] extended this concept to interval valued numbers as:

**Definition** **3.** Ref. [4] An interval valued intuitionistic set (IVIFS) A defined over the universal set X is defined as $A=\left\{\langle x,\left[\zeta_{A}^{L}\left(x\right),\zeta_{A}^{U}\left(x\right)\right],\left[\vartheta_{A}^{L}\left(x\right),\vartheta_{A}^{U}\left(x\right)\right]\rangle \right\}$, such that $\left[\zeta_{A}^{L}\left(x\right),\zeta_{A}^{U}\left(x\right)\right],\left[\vartheta_{A}^{L}\left(x\right),\vartheta_{A}^{U}\left(x\right)\right]\subseteq \left[0,1\right]$ and $0\leq \zeta_{A}^{U}\left(x\right)+\vartheta_{A}^{U}\left(x\right)\leq 1$ for each x. For convenience, we denote this pair as $\alpha =\left(\left[\zeta_{A}^{L},\zeta_{A}^{U}\right],\left[\vartheta_{A}^{L},\vartheta_{A}^{U}\right]\right)$ and called as an interval-valued intuitionistic fuzzy number (IVIFN). Furthermore, based on the operations of IVIFNs, Xu [41] defined some operations of its as follows.

**Definition** **4.** *Ref. [41] Let $\alpha =\left(\left[\zeta^{L},\zeta^{U}\right],\left[\vartheta^{L},\vartheta^{U}\right]\right)$, $\alpha_{1}=\left(\left[\zeta_{1}^{L},\zeta_{1}^{U}\right],\left[\vartheta_{1}^{L},\vartheta_{1}^{U}\right]\right)$ and $\alpha_{2}=\left(\left[\zeta_{2}^{L},\zeta_{2}^{U}\right],\left[\vartheta_{2}^{L},\vartheta_{2}^{U}\right]\right)$ be three IVIFNs and ξ>0 be a real number, then the following operational laws are valid:*

*(i)*  $\alpha_{1}⊕\alpha_{2}=\left(\left[1-\left(1-\zeta_{1}^{L}\right)\left(1-\zeta_{2}^{L}\right),1-\left(1-\zeta_{1}^{U}\right)\left(1-\zeta_{2}^{U}\right)\right],\left[\vartheta_{1}^{L}\vartheta_{2}^{L},\vartheta_{1}^{U}\vartheta_{2}^{U}\right]\right)$,
*(ii)*  $\alpha_{1}⊗\alpha_{2}=\left(\left[\zeta_{1}^{L}\zeta_{2}^{L},\zeta_{1}^{U}\zeta_{2}^{U}\right],\left[1-\left(1-\vartheta_{1}^{L}\right)\left(1-\vartheta_{2}^{L}\right),1-\left(1-\vartheta_{1}^{U}\right)\left(1-\vartheta_{2}^{U}\right)\right]\right)$,
*(iii)*  $\xi \alpha =\left(\left[1-{\left(1-\zeta^{L}\right)}^{\xi },1-{\left(1-\zeta^{U}\right)}^{\xi }\right],\left[{\left(\vartheta^{L}\right)}^{\xi },{\left(\vartheta^{U}\right)}^{\xi }\right]\right)$,
*(iv)*  ${\left(\alpha \right)}^{\xi }=\left(\left[{\left(\zeta^{L}\right)}^{\xi },{\left(\zeta^{U}\right)}^{\xi }\right],\left[1-{\left(1-\vartheta^{L}\right)}^{\xi },1-{\left(1-\vartheta^{U}\right)}^{\xi }\right]\right)$.

**Definition** **5.** *Ref. [38] A cubic fuzzy set (CFS) ‘A’ is defined over universal set X as*

(1) $$\begin{array}{c} A=\left\{(x,A_{F}\left(x\right),\lambda_{F}\left(x\right))|x\in X\right\} \end{array}$$

*where, $A_{F}\left(x\right)=\left[A^{L}\left(x\right),A^{U}\left(x\right)\right]$ is an interval-valued FS and $\lambda_{F}\left(x\right)$ represents a FS in X.*

**Definition** **6.** *For any $A_{i}=\langle A_{i},\lambda_{i}\rangle$ where i∈Λ, we have [38]*

*(i)*  P-union: ∪Pi∈ΛAi=∪i∈ΛAi,∨i∈Λλi.
*(ii)*  P-intersection: ∩Pi∈ΛAi=∩i∈ΛAi,∧i∈Λλi.
*(iii)*  R-union: ∪Ri∈ΛAi=∪i∈ΛAi,∧i∈Λλi.
*(iv)*  R-intersection: ∩Ri∈ΛAi=∩i∈ΛAi,∨i∈Λλi.

**Definition** **7.** *Ref. [30] For p,q≥0 and $a_{i}$(i=1,2,…,n) a collection of non-negative numbers. If*

(2) $$\begin{array}{c} {\mathrm{BM}}^{p,q}\left(a_{1},a_{2},\ldots ,a_{n}\right)={\left(\frac{1}{n(n-1)}\sum_{\begin{array}{c} i,j=1\\ i\neq j \end{array}}^{n}{a_{i}}^{p}{a_{j}}^{q}\right)}^{\frac{1}{p+q}} \end{array}$$

*then ${\mathrm{BM}}^{p,q}$ is called the Bonferroni mean (BM) operator.*

Xu and Yager [32] extended this BM to intuitionistic fuzzy environment and gave the following concepts.

**Definition** **8.** *Ref. [32] Let $\delta_{i}$ be a set of non-negative intuitionistic fuzzy numbers. The intuitionistic fuzzy Bonferroni mean (IFBM), the intuitionistic fuzzy weighted Bonferroni mean (IFWBM) are respectively, defined as*

IFBMp,q(α1,α2,…,αn)=1n(n−1)⨁i,j=1i≠jnαip⊗αjq1/(p+q)

*and*

IFWBMp,q(α1,α2,…,αn)=1n(n−1)⨁i,j=1i≠jnωiαip⊗ωjαjq1/(p+q)

## 3. Cubic Intuitionistic Fuzzy Sets and the Aggregation Operators

In this section, we have defined some basic operational laws between the pairs of the R-order CIFNs, denoted by Ω, and hence based on it, some series of Bonferroni mean operators have been proposed.

### 3.1. Cubic Intuitionistic Fuzzy Set

**Definition** **9.** *A CIFS A defined over the universal set X is an ordered pair which is defined as follows*

(3) $$\begin{array}{c} A=\{\langle x,A(x),\lambda (x)\rangle |x\in X\} \end{array}$$

*where $A=\{x,\langle \left[\zeta_{A}^{L}\left(x\right),\zeta_{A}^{U}\left(x\right)\right],\left[\vartheta_{A}^{L}\left(x\right),\vartheta_{A}^{U}\left(x\right)\right]\rangle ,|x\in X\}$ represents the IVIFS defined on X while $\lambda \left(x\right)=\{x,\langle \zeta_{A}\left(x\right),\vartheta_{A}\left(x\right)\rangle |x\in X\}$ represents an IFS such that $0\leq \zeta_{A}^{L}\left(x\right)\leq \zeta_{A}^{U}\left(x\right)\leq 1$, $0\leq \vartheta_{A}^{L}\left(x\right)\leq \vartheta_{A}^{U}\left(x\right)\leq 1$ and $0\leq \zeta_{A}^{U}\left(x\right)+\vartheta_{A}^{U}\left(x\right)\leq 1$. Also, $0\leq \zeta_{A}\left(x\right),\vartheta_{A}\left(x\right)\leq 1$ and $\zeta_{A}\left(x\right)+\vartheta_{A}\left(x\right)\leq 1$. For the sake of simplicity, we denote these pairs as $A=\langle A,\lambda \rangle$, where $A=\langle \left[\zeta_{A}^{L},\zeta_{A}^{U}\right],\left[\vartheta_{A}^{L},\vartheta_{A}^{U}\right]\rangle$ and $\lambda =\langle \zeta_{A},\vartheta_{A}\rangle$ and called as cubic intuitionistic fuzzy number (CIFN).*

**Definition** **10.** A CIFS A defined in Equation (3) is said to be Internal CIFS if $\zeta_{A}\left(x\right)\in \left[\zeta_{A}^{L}\left(x\right),\zeta_{A}^{U}\left(x\right)\right]$ and $\vartheta_{A}\left(x\right)\in \left[\vartheta_{A}^{L}\left(x\right),\vartheta_{A}^{U}\left(x\right)\right]$ for all x∈X, otherwise called as External Cubic Intuitionistic fuzzy set.

**Definition** **11.** *For a family of CIFS $\left\{A_{i},i\in \Lambda \right\}$, we have*

*(a)*  (P-union): ⋃Pi∈ΛAi=(〈[supi∈ΛζiL,supi∈ΛζiU], $[\underset{i\in \Lambda }{\inf}\vartheta_{i}^{L},\underset{i\in \Lambda }{\inf}\vartheta_{i}^{U}]\rangle$, $\langle \underset{i\in \Lambda }{\sup}\zeta_{i}$, $\underset{i\in \Lambda }{\inf}\vartheta_{i}\rangle )$.
*(b)*  (P-intersection): ⋂Pi∈ΛAi=(〈[infi∈ΛζiL,infi∈ΛζiU], $[\underset{i\in \Lambda }{\sup}\vartheta_{i}^{L},\underset{i\in \Lambda }{\sup}\vartheta_{i}^{U}]\rangle$, $\langle \underset{i\in \Lambda }{\inf}\zeta_{i}$, $\underset{i\in \Lambda }{\sup}\vartheta_{i}\rangle )$.
*(c)*  (R-union):⋃Ri∈ΛAi=(〈[supi∈ΛζiL,supi∈ΛζiU], $[\underset{i\in \Lambda }{\inf}\vartheta_{i}^{L},\underset{i\in \Lambda }{\inf}\vartheta_{i}^{U}]\rangle$, $\langle \underset{i\in \Lambda }{\inf}\zeta_{i}$, $\underset{i\in \Lambda }{\sup}\vartheta_{i}\rangle )$.
*(d)*  (R-intersection): ⋂Ri∈ΛAi=(〈[infi∈ΛζiL,infi∈ΛζiU], $[\underset{i\in \Lambda }{\sup}\vartheta_{i}^{L},\underset{i\in \Lambda }{\sup}\vartheta_{i}^{U}]\rangle$, $\langle \underset{i\in \Lambda }{\sup}\zeta_{i}$, $\underset{i\in \Lambda }{\inf}\vartheta_{i}\rangle )$.

**Definition** **12.** *Let $\delta_{i}=(\langle \left[\zeta_{i}^{L},\zeta_{i}^{U}\right],\left[\vartheta_{i}^{L},\vartheta_{i}^{U}\right]\rangle ,\langle \zeta_{i},\vartheta_{i}\rangle )$ where i=(1,2) be two CIFNs in X. Then we define:*

*(a)*  (Equality) $\delta_{1}=\delta_{2}$ if and only if $\left[\zeta_{1}^{L},\zeta_{1}^{U}\right]=\left[\zeta_{2}^{L},\zeta_{2}^{U}\right]$, $\left[\vartheta_{1}^{L},\vartheta_{1}^{U}\right]=\left[\vartheta_{2}^{L},\vartheta_{2}^{U}\right]$, $\zeta_{1}$ = $\zeta_{2}$ and $\vartheta_{1}$ = $\vartheta_{2}$.
*(b)*  (P-order) $\delta_{1}\subseteq_{P}\delta_{2}$ if $\left[\zeta_{1}^{L},\zeta_{1}^{U}\right]\subseteq \left[\zeta_{2}^{L},\zeta_{2}^{U}\right]$,$\left[\vartheta_{1}^{L},\vartheta_{1}^{U}\right]⊇\left[\vartheta_{2}^{L},\vartheta_{2}^{U}\right]$, $\zeta_{1}\leq \zeta_{2}$ and $\vartheta_{1}\geq \vartheta_{2}$
*(c)*  (R-order) $\delta_{1}\subseteq_{R}\delta_{2}$ if $\left[\zeta_{1}^{L},\zeta_{1}^{U}\right]\subseteq \left[\zeta_{2}^{L},\zeta_{2}^{U}\right]$,$\left[\vartheta_{1}^{L},\vartheta_{1}^{U}\right]⊇\left[\vartheta_{2}^{L},\vartheta_{2}^{U}\right]$, $\zeta_{1}\geq \zeta_{2}$ and $\vartheta_{1}\leq \vartheta_{2}$

**Definition** **13.** *The score function to rank the CIFN $\delta_{i}=\left(\langle \left[\zeta_{i}^{L},\zeta_{i}^{U}\right],\left[\vartheta_{i}^{L},\vartheta_{i}^{U}\right]\rangle ,\langle \zeta_{i},\vartheta_{i}\rangle \right)$ is defined under R-order as*

(4) $$\begin{array}{c} \mathrm{Sc}\left(\delta_{i}\right)=\frac{\zeta_{i}^{L}+\zeta_{i}^{U}-\vartheta_{i}^{L}-\vartheta_{i}^{U}}{2}+\left(\vartheta_{i}-\zeta_{i}\right) \end{array}$$

*while for P-order as*

(5) $$\begin{array}{c} \mathrm{Sc}\left(\delta_{i}\right)=\frac{\zeta_{i}^{L}+\zeta_{i}^{U}-\vartheta_{i}^{L}-\vartheta_{i}^{U}}{2}+\left(\zeta_{i}-\vartheta_{i}\right) \end{array}$$

*Also, an accuracy function is defined as*

(6) $$\begin{array}{c} H\left(\delta_{i}\right)=\frac{\zeta_{i}^{L}+\zeta_{i}^{U}+\vartheta_{i}^{L}+\vartheta_{i}^{U}}{2}+\left(\zeta_{i}+\vartheta_{i}\right) \end{array}$$

It is evident that $-2\leq \mathrm{Sc}(\delta_{i})\leq 2$ and $0\leq H(\delta_{i})\leq 2$.

**Definition** **14.** *For any two CIFNs $\delta_{i}$, the following comparison rule has been defined*

*(i)*  if $Sc\left(\delta_{1}\right)>Sc\left(\delta_{2}\right)$ then $\delta_{1}$ is preferable over $\delta_{2}$ and is denoted by $\delta_{1}≻\delta_{2}$.
*(ii)*  *if $Sc\left(\delta_{1}\right)=Sc\left(\delta_{2}\right)$*
  *(a)*  *if $H\left(\delta_{1}\right)>H\left(\delta_{2}\right)$ then $\delta_{1}≻\delta_{2}$.*
  *(b)*  *if $H\left(\delta_{1}\right)=H\left(\delta_{2}\right)$ then $\delta_{1}∼\delta_{2}$, where ∼ represent “equivalent to”.*

**Theorem** **1.** *For CIFSs $A=\langle A,\lambda \rangle ,B=\langle B,\mu \rangle ,\;C=\langle C,\gamma \rangle$ and $D=\langle D,\rho \rangle$, where A,B,C and D are IVIFSs and λ,μ,γ and ρ are IFSs in X, we have*

*(i)*  if $A\subseteq_{P}B$ and $B\subseteq_{P}C$ then $A\subseteq_{P}C$,
*(ii)*  if $A\subseteq_{P}B$ then $B^{c}\subseteq_{P}A^{c}$,
*(iii)*  if $A\subseteq_{P}B$ and $A\subseteq_{P}C$ then $A\subseteq_{P}B\cap_{P}C$,
*(iv)*  if $A\subseteq_{P}B$ and $C\subseteq_{P}B$ then $A\cup_{P}C\subseteq_{P}B$,
*(v)*  if $A\subseteq_{P}B$ and $C\subseteq_{P}D$ then $A\cup_{P}C\subseteq_{P}B\cup_{P}D$ and $A\cap_{P}C\subseteq_{P}B\cap_{P}D$,
*(vi)*  if $A\subseteq_{R}B$ and if $B\subseteq_{R}C$ then $A\subseteq_{R}C$,
*(vii)*  if $A\subseteq_{R}B$ then $B^{c}\subseteq_{R}A^{c}$,
*(viii)*  if $A\subseteq_{R}B$ and $A\subseteq_{R}C$ then $A\subseteq_{R}B\cap_{R}C$,
*(ix)*  if $A\subseteq_{R}B$ and $C\subseteq_{R}B$ then $A\cup_{R}C\subseteq_{R}B$,
*(x)*  if $A\subseteq_{R}B$ and $C\subseteq_{R}D$ then $A\cup_{R}C\subseteq_{R}B\cup_{R}D$ and $A\cap_{R}C\subseteq_{R}B\cap_{R}D$,

**Proof.**  Straightforward, so proof is omitted. ☐

**Definition** **15.** *Let $\delta =(\langle [\zeta^{L},\zeta^{U}]$, $[\vartheta^{L},\vartheta^{U}]\rangle$, $\langle \zeta ,\vartheta \rangle )$, $\delta_{i}=(\langle \left[\zeta_{i}^{L},\zeta_{i}^{U}\right],\left[\vartheta_{i}^{L},\vartheta_{i}^{U}\right]\rangle ,\langle \zeta_{i},\vartheta_{i}\rangle )$, (i=1,2,…,n) be the collections of CIFNs, and ξ>0 be a real number then the operational laws on these CIFNs are defined as below:*

*(i)*  $\delta_{1}⊕\delta_{2}=\left(\langle \left[1-\prod_{i=1}^{2}\left(1-\zeta_{i}^{L}\right),1-\prod_{i=1}^{2}\left(1-\zeta_{i}^{U}\right)\right],\left[\prod_{i=1}^{2}\vartheta_{i}^{L},\prod_{i=1}^{2}\vartheta_{i}^{U}\right]\rangle ,\langle \prod_{i=1}^{2}\zeta_{i},1-\prod_{i=1}^{2}\left(1-\vartheta_{i}\right)\rangle \right)$
*(ii)*  $\delta_{1}⊗\delta_{2}=\left(\langle \left[\prod_{i=1}^{2}\zeta_{i}^{L},\prod_{i=1}^{2}\zeta_{i}^{U}\right],\left[1-\prod_{i=1}^{2}\left(1-\vartheta_{i}^{L}\right),1-\prod_{i=1}^{2}\left(1-\vartheta_{i}^{U}\right)\right]\rangle ,\langle 1-\prod_{i=1}^{2}\left(1-\zeta_{i}\right),\prod_{i=1}^{2}\vartheta_{i}\rangle \right)$
*(iii)*  $\xi \delta =\left(\langle \left[1-{\left(1-\zeta^{L}\right)}^{\xi },1-{\left(1-\zeta^{U}\right)}^{\xi }\right],\left[{\left(\vartheta^{L}\right)}^{\xi },{\left(\vartheta^{U}\right)}^{\xi }\right]\rangle ,\langle {\left(\zeta \right)}^{\xi },1-{\left(1-\vartheta \right)}^{\xi }\rangle \right)$
*(iv)*  $\delta^{\xi }=\left(\langle \left[{\left(\zeta^{L}\right)}^{\xi },{\left(\zeta^{U}\right)}^{\xi }\right],\left[1-{\left(1-\vartheta^{L}\right)}^{\xi },1-{\left(1-\vartheta^{U}\right)}^{\xi }\right]\rangle ,\langle 1-{\left(1-\zeta \right)}^{\xi },{\left(\vartheta \right)}^{\xi }\rangle \right)$

**Theorem** **2.** For two CIFNs $\delta_{1}$ and $\delta_{2}$, ξ>0 be a real number then $\delta_{1}⊕\delta_{2}$, $\delta_{1}⊗\delta_{2}$, $\xi \delta_{1}$ and $\delta_{1}^{\xi }$ are also CIFNs.

**Proof.**  Since $\delta_{1}=\langle \langle \left[\zeta_{1}^{L},\zeta_{1}^{U}\right],\left[\vartheta_{1}^{L},\vartheta_{1}^{U}\right]\rangle ,\langle \zeta_{1},\vartheta_{1}\rangle \rangle$ and $\delta_{2}=\langle \langle \left[\zeta_{2}^{L},\zeta_{2}^{U}\right],\left[\vartheta_{2}^{L},\vartheta_{2}^{U}\right]\rangle ,\langle \zeta_{2},\vartheta_{2}\rangle \rangle$ are two CIFNs such that $0\leq \zeta_{1}^{L},\zeta_{2}^{L},\zeta_{1}^{U},\zeta_{2}^{U}$, $\vartheta_{1}^{L},\vartheta_{2}^{L},\vartheta_{1}^{U},\vartheta_{2}^{U}\leq 1$ and $\zeta_{1}^{U}+\vartheta_{1}^{U}\leq 1$, $\zeta_{2}^{U}+\vartheta_{2}^{U}\leq 1$ which implies that $0\leq \left(1-\zeta_{1}^{L}\right)\left(1-\zeta_{2}^{L}\right)\leq 1$ and hence $0\leq \zeta_{1}^{L}+\zeta_{2}^{L}-\zeta_{1}^{L}\zeta_{2}^{L}\leq 1$. Similarly, we can prove that $0\leq \zeta_{1}^{U}+\zeta_{2}^{U}-\zeta_{1}^{U}\zeta_{2}^{U}\leq 1$, $0\leq \vartheta_{1}^{L}\vartheta_{2}^{L}\leq 1$ and $0\leq \vartheta_{1}^{U}\vartheta_{2}^{U}\leq 1$. Also, $0\leq \zeta_{1},\zeta_{2},\vartheta_{1},\vartheta_{2}\leq 1$ and $\zeta_{1}+\vartheta_{1}\leq 1$, $\zeta_{2}+\vartheta_{2}\leq 1$ which implies that $\zeta_{1}\zeta_{2}\leq 1$ and $0\leq \vartheta_{1}+\vartheta_{2}-\vartheta_{1}\vartheta_{2}\leq 1$. Finally, we have $\zeta_{1}^{U}+\zeta_{2}^{U}-\zeta_{1}^{U}\zeta_{2}^{U}+\vartheta_{1}^{U}\vartheta_{2}^{U}=1-\left(1-\zeta_{1}^{U}\right)\left(1-\zeta_{2}^{U}\right)+\vartheta_{1}^{U}\vartheta_{2}^{U}\leq 1-\vartheta_{1}^{U}\vartheta_{2}^{U}+\vartheta_{1}^{U}\vartheta_{2}^{U}\leq 1$ and $\zeta_{1}\zeta_{2}+\vartheta_{1}+\vartheta_{2}-\vartheta_{1}\vartheta_{2}=\zeta_{1}\zeta_{2}+1-\left(1-\vartheta_{1}\right)\left(1-\vartheta_{2}\right)\leq \zeta_{1}\zeta_{2}+1-\zeta_{1}\zeta_{2}\leq 1$. Therefore, $\delta_{1}⊕\delta_{2}$ is CIFN.

Further, for any positive number ξ and CIFN δ, we have $0\leq \zeta_{1}^{\xi }\leq 1$ , $0\leq 1-{\left(1-\vartheta_{1}\right)}^{\xi }\leq 1$, $0\leq {\left(\vartheta_{1}^{L}\right)}^{\xi },{\left(\vartheta_{1}^{U}\right)}^{\xi }\leq 1$ and $0\leq 1-{\left(1-\zeta_{1}^{L}\right)}^{\xi },1-{\left(1-\zeta_{1}^{U}\right)}^{\xi }\leq 1$. Thus, $\xi \delta_{1}$ is also CIFN. Similarly, we can prove that $\delta_{1}⊗\delta_{2}$ and $\delta_{1}^{\xi }$ are also CIFNs. ☐

### 3.2. Cubic Intuitionistic Fuzzy Bonferroni Mean Operator

**Definition** **16.** *A cubic intuitionistic fuzzy Bonferroni mean (CIFBM) operator is a mapping $\mathrm{CIFBM}:\;\Omega^{n}\to \Omega$ defined on the collection of CIFNs $\delta_{i}$, and is given by*

(7) CIFBMp,q(δ1,δ2,…,δn)=1n(n−1)⨁i,j=1i≠jn(δip⊗δjq)1p+q

*where p,q>0 are the real numbers.*

**Theorem** **3.** *The aggregated value by using CIFBM operator for CIFNs $\delta_{i}=\left(\langle \left[\zeta_{i}^{L},\zeta_{i}^{U}\right],\left[\vartheta_{i}^{L},\vartheta_{i}^{U}\right]\rangle ,\langle \zeta_{i},\vartheta_{i}\rangle \right)$ is still CIFN and is given by*

(8) CIFBMp,q(δ1,δ2,…,δn)=〈1−∏i,j=1i≠jn1−(ζiL)p(ζjL)q1n(n−1)1p+q,1−∏i,j=1i≠jn1−(ζiU)p(ζjU)q1n(n−1)1p+q,1−1−∏i,j=1i≠jn1−(1−ϑiL)p(1−ϑjL)q1n(n−1)1p+q,1−1−∏i,j=1i≠jn1−(1−ϑiU)p(1−ϑjU)q1n(n−1)1p+q〉,1−1−∏i,j=1i≠jn1−(1−ζi)p(1−ζj)q1n(n−1)1p+q,1−∏i,j=1i≠jn1−(ϑi)p(ϑj)q1n(n−1)1p+q

**Proof.**  For any two positive real numbers p,q and CIFNs $\delta_{i}$, $\delta_{j}$, we have from the basic operational laws between CIFNs given in Definition 15,

(9) $$\begin{array}{c} \delta_{i}^{p}=\left(\left[{\left(\zeta_{i}^{L}\right)}^{p},{\left(\zeta_{i}^{U}\right)}^{p}\right],\left[1-{\left(1-\vartheta_{i}^{L}\right)}^{p},1-{\left(1-\vartheta_{i}^{U}\right)}^{p}\right]\rangle ,\langle 1-{\left(1-\zeta_{i}\right)}^{p},{\left(\vartheta_{i}\right)}^{p}\right) \end{array}$$

(10) $$\begin{array}{c} \mathrm{and}\;\delta_{j}^{q}=\left(\left[{\left(\zeta_{j}^{L}\right)}^{q},{\left(\zeta_{j}^{U}\right)}^{q}\right],\left[1-{\left(1-\vartheta_{j}^{L}\right)}^{q},1-{\left(1-\vartheta_{j}^{U}\right)}^{q}\right]\rangle ,\langle 1-{\left(1-\zeta_{j}\right)}^{q},{\left(\vartheta_{j}\right)}^{q}\right) \end{array}$$

Therefore,

(11) $$\begin{array}{ccc} \delta_{i}^{p}⊗\delta_{j}^{q} & = & \left(\begin{array}{c} \langle \left[{\left(\zeta_{i}^{L}\right)}^{p}{\left(\zeta_{j}^{L}\right)}^{q},{\left(\zeta_{i}^{U}\right)}^{p}{\left(\zeta_{j}^{U}\right)}^{q}\right],[1-{\left(1-\vartheta_{i}^{L}\right)}^{p}{\left(1-\vartheta_{j}^{L}\right)}^{q},\\ 1-{\left(1-\vartheta_{i}^{U}\right)}^{p}{\left(1-\vartheta_{j}^{U}\right)}^{q}]\rangle ,\langle 1-{\left(1-\zeta_{i}\right)}^{p}{\left(1-\zeta_{j}\right)}^{q},{\left(\vartheta_{i}\right)}^{p}{\left(\vartheta_{j}\right)}^{q}\rangle \end{array}\right) \end{array}$$

Firstly, we prove

(12) ⨁i,j=1i≠jn(δip⊗δjq)=〈[1−∏i,j=1i≠jn1−(ζiL)p(ζjL)q,1−∏i,j=1i≠jn1−(ζiU)p(ζjU)q],[∏i,j=1i≠jn1−(1−ϑiL)p(1−ϑjL)q,∏i,j=1i≠jn1−(1−ϑiU)p(1−ϑjU)q]〉,∏i,j=1i≠jn1−(1−ζi)p(1−ζj)q,1−∏i,j=1i≠jn1−(ϑi)p(ϑj)q

by induction on *n*.

For n=2 we get,

(13) ⨁i,j=1i≠j2(δip⊗δjq)=〈1−∏i,j=1i≠j21−(ζiL)p(ζjL)q,1−∏i,j=1i≠j21−(ζiU)p(ζjU)q,∏i,j=1i≠j21−(1−ϑiL)p(1−ϑjL)q,∏i,j=1i≠j21−(1−ϑiU)p(1−ϑjU)q〉,∏i,j=1i≠j21−(1−ζi)p(1−ζj)q,1−∏i,j=1i≠j21−(ϑi)p(ϑj)q

Thus, it holds for n=2. Assuming result is true for n=k i.e.,

(14) ⨁i,j=1i≠jk(δip⊗δjq)=〈[1−∏i,j=1i≠jk1−(ζiL)p(ζjL)q,1−∏i,j=1i≠jk1−(ζiU)p(ζjU)q],[∏i,j=1i≠jk1−(1−ϑiL)p(1−ϑjL)q,∏i,j=1i≠jk1−(1−ϑiU)p(1−ϑjU)q]〉,∏i,j=1i≠jk1−(1−ζi)p(1−ζj)q,1−∏i,j=1i≠jk1−(ϑi)p(ϑj)q

Now, for n=k+1, we have

(15) ⨁i,j=1i≠jk+1δip⊗δjq=⨁i,j=1i≠jkδip⊗δjq⊕⨁i=1kδip⊗δk+1q⊕⨁j=1kδk+1p⊗δjq

Now, we shall prove

(16) $$\begin{array}{ccc} \overset{k}{\underset{i=1}{⨁}}\left(\delta_{i}^{p}⊗\delta_{k+1}^{q}\right) & = & \left(\begin{array}{c} \langle \left[1-\prod_{i=1}^{k}\left(1-{\left(\zeta_{i}^{L}\right)}^{p}{\left(\zeta_{k+1}^{L}\right)}^{q}\right),1-\prod_{i=1}^{k}\left(1-{\left(\zeta_{i}^{U}\right)}^{p}{\left(\zeta_{k+1}^{U}\right)}^{q}\right)\right],\\ \left[\prod_{i=1}^{k}\left(1-{\left(1-\vartheta_{i}^{L}\right)}^{p}{\left(1-\vartheta_{k+1}^{L}\right)}^{q}\right),\prod_{i=1}^{k}\left(1-{\left(1-\vartheta_{i}^{U}\right)}^{p}{\left(1-\vartheta_{k+1}^{U}\right)}^{q}\right)\right]\rangle ,\\ \langle \prod_{i=1}^{k}\left(1-{\left(1-\zeta_{i}\right)}^{p}{\left(1-\zeta_{k+1}\right)}^{q}\right),1-\prod_{i=1}^{k}\left(1-{\left(\vartheta_{i}\right)}^{p}{\left(\vartheta_{k+1}\right)}^{q}\right)\rangle \end{array}\right) \end{array}$$

Again, for k=2, using Equation (11), we have

(17) $$\begin{array}{ccc} \delta_{i}^{p}⊗\delta_{2+1}^{q} & = & \left(\begin{array}{c} \langle \left[{\left(\zeta_{i}^{L}\right)}^{p}{\left(\zeta_{2+1}^{L}\right)}^{q},{\left(\zeta_{i}^{U}\right)}^{p}{\left(\zeta_{2+1}^{U}\right)}^{q}\right],[1-{\left(1-\vartheta_{i}^{L}\right)}^{p}{\left(1-\vartheta_{2+1}^{L}\right)}^{q},\\ 1-{\left(1-\vartheta_{i}^{U}\right)}^{p}{\left(1-\vartheta_{2+1}^{U}\right)}^{q}]\rangle ,\langle 1-{\left(1-\zeta_{i}\right)}^{p}{\left(1-\zeta_{2+1}\right)}^{q},{\left(\vartheta_{i}\right)}^{p}{\left(\vartheta_{2+1}\right)}^{q}\rangle \end{array}\right) \end{array}$$

and thus,

(18) $$\begin{array}{ccc} \overset{2}{\underset{i=1}{⨁}}\left(\delta_{i}^{p}⊗\delta_{2+1}^{q}\right) & = & \left(\delta_{1}^{p}⊗\delta_{2+1}^{q}\right)⊕\left(\delta_{2}^{p}⊗\delta_{2+1}^{q}\right)\\ & = & \left(\begin{array}{c} \langle \left[1-\prod_{i=1}^{2}\left(1-{\left(\zeta_{i}^{L}\right)}^{p}{\left(\zeta_{3}^{L}\right)}^{q}\right),1-\prod_{i=1}^{2}\left(1-{\left(\zeta_{i}^{U}\right)}^{p}{\left(\zeta_{3}^{U}\right)}^{q}\right)\right],\\ \left[\prod_{i=1}^{2}\left(1-{\left(1-\vartheta_{i}^{L}\right)}^{p}{\left(1-\vartheta_{3}^{L}\right)}^{q}\right),\prod_{i=1}^{2}\left(1-{\left(1-\vartheta_{i}^{U}\right)}^{p}{\left(1-\vartheta_{3}^{U}\right)}^{q}\right)\right]\rangle ,\\ \langle \prod_{i=1}^{2}\left(1-{\left(1-\zeta_{i}\right)}^{p}{\left(1-\zeta_{3}\right)}^{q}\right),1-\prod_{i=1}^{2}\left(1-{\left(\vartheta_{i}\right)}^{p}{\left(\vartheta_{3}\right)}^{q}\right)\rangle \end{array}\right) \end{array}$$

If Equation (16) holds for $k=k_{0}$ i.e.,

(19) $$\begin{array}{ccc} \overset{k_{0}}{\underset{i=1}{⨁}}\left(\delta_{i}^{p}⊗\delta_{k_{0}+1}^{q}\right) & = & \left(\begin{array}{c} \langle \left[1-\prod_{i=1}^{k_{0}}\left(1-{\left(\zeta_{i}^{L}\right)}^{p}{\left(\zeta_{k_{0}+1}^{L}\right)}^{q}\right),1-\prod_{i=1}^{k_{0}}\left(1-{\left(\zeta_{i}^{U}\right)}^{p}{\left(\zeta_{k_{0}+1}^{U}\right)}^{q}\right)\right],\\ \left[\prod_{i=1}^{k_{0}}\left(1-{\left(1-\vartheta_{i}^{L}\right)}^{p}{\left(1-\vartheta_{k_{0}+1}^{L}\right)}^{q}\right),\prod_{i=1}^{k_{0}}\left(1-{\left(1-\vartheta_{i}^{U}\right)}^{p}{\left(1-\vartheta_{k_{0}+1}^{U}\right)}^{q}\right)\right]\rangle ,\\ \langle \prod_{i=1}^{k_{0}}\left(1-{\left(1-\zeta_{i}\right)}^{p}{\left(1-\zeta_{k_{0}+1}\right)}^{q}\right),1-\prod_{i=1}^{k_{0}}\left(1-{\left(\vartheta_{i}\right)}^{p}{\left(\vartheta_{k_{0}+1}\right)}^{q}\right)\rangle \end{array}\right) \end{array}$$

then, for $k=k_{0}+1$, using Definition 15 we have:

(20) $$\begin{array}{ccc} \overset{k_{0}+1}{\underset{i=1}{⨁}}\left(\delta_{i}^{p}⊗\delta_{k_{0}+2}^{q}\right) & = & \overset{k_{0}}{\underset{i=1}{⨁}}\left(\delta_{i}^{p}⊗\delta_{k_{0}+2}^{q}\right)⊕\left(\delta_{k_{0}+1}^{p}⊗\delta_{k_{0}+2}^{q}\right)\\ & = & \left(\begin{array}{c} \langle \left[1-\prod_{i=1}^{k_{0}+1}\left(1-{\left(\zeta_{i}^{L}\right)}^{p}{\left(\zeta_{k_{0}+2}^{L}\right)}^{q}\right),\left(1-{\left(\zeta_{i}^{U}\right)}^{p}{\left(\zeta_{k_{0}+2}^{U}\right)}^{q}\right)\right],\\ \left[\prod_{i=1}^{k_{0}+1}\left(1-{\left(1-\vartheta_{i}^{L}\right)}^{p}{\left(1-\vartheta_{k_{0}+2}^{L}\right)}^{q}\right),\prod_{i=1}^{k_{0}+1}\left(1-{\left(1-\vartheta_{i}^{U}\right)}^{p}{\left(1-\vartheta_{k_{0}+2}^{U}\right)}^{q}\right)\right]\rangle ,\\ \langle \prod_{i=1}^{k_{0}+1}\left(1-{\left(1-\zeta_{i}\right)}^{p}{\left(1-\zeta_{k_{0}+2}\right)}^{q}\right),1-\prod_{i=1}^{k_{0}+1}\left(1-{\left(\vartheta_{i}\right)}^{p}{\left(\vartheta_{k_{0}+2}\right)}^{q}\right)\rangle \end{array}\right) \end{array}$$

and hence Equation (16) holds for $k=k_{0}+1$. Thus, it holds true for every *k*. Similarly,

(21) $$\begin{array}{ccc} \overset{k}{\underset{j=1}{⨁}}\left(\delta_{k+1}^{p}⊗\delta_{j}^{q}\right) & = & \left(\begin{array}{c} \langle \left[1-\prod_{j=1}^{k}\left(1-{\left(\zeta_{k+1}^{L}\right)}^{p}{\left(\zeta_{j}^{L}\right)}^{q}\right),1-\prod_{j=1}^{k}\left(1-{\left(\zeta_{k+1}^{U}\right)}^{p}{\left(\zeta_{j}^{U}\right)}^{q}\right)\right],\\ \left[\prod_{j=1}^{k}\left(1-{\left(1-\vartheta_{k+1}^{L}\right)}^{p}{\left(1-\vartheta_{j}^{L}\right)}^{q}\right),\prod_{j=1}^{k}\left(1-{\left(1-\vartheta_{k+1}^{U}\right)}^{p}{\left(1-\vartheta_{j}^{U}\right)}^{q}\right)\right]\rangle ,\\ \langle \prod_{j=1}^{k}\left(1-{\left(1-\zeta_{k+1}\right)}^{p}{\left(1-\zeta_{j}\right)}^{q}\right),1-\prod_{j=1}^{k}\left(1-{\left(\vartheta_{k+1}\right)}^{p}{\left(\vartheta_{j}\right)}^{q}\right)\rangle \end{array}\right) \end{array}$$

Therefore, by using Equations (14), (16) and (21), Equation (15) becomes

⨁i,j=1i≠jkδip⊗δjq=〈[1−∏i,j=1i≠jk1−(ζiL)p(ζjL)q,1−∏i,j=1i≠jk1−(ζiU)p(ζjU)q],[∏i,j=1i≠jk1−(1−ϑiL)p(1−ϑjL)q,∏i,j=1i≠jk1−(1−ϑiU)p(1−ϑjU)q]〉,∏i,j=1i≠jk1−(1−ζi)p(1−ζj)q,1−∏i,j=1i≠jk1−(ϑi)p(ϑj)q⊕〈1−∏i=1k1−(ζiL)p(ζk+1L)q,1−∏i=1k1−(ζiU)p(ζk+1U)q,∏i=1k1−1−ϑiLp1−ϑk+1Lq,∏i=1k1−1−ϑiUp1−ϑk+1Uq〉,∏i=1k1−1−ζip1−ζk+1q,1−∏i=1k1−(ϑi)p(ϑk+1)q⊕〈1−∏j=1k1−(ζk+1L)p(ζjL)q,1−∏j=1k1−(ζk+1U)p(ζjU)q,∏i=1k1−1−ϑk+1Lp1−ϑjLq,∏i=1k1−1−ϑk+1Up1−ϑjUq〉,∏i=1k1−1−ζk+1p1−ζjq,1−1−ϑk+1p1−ϑjq〉=〈[1−∏i,j=1i≠jk+11−(ζiL)p(ζjL)q,1−∏i,j=1i≠jk+11−(ζiU)p(ζjU)q],[∏i,j=1i≠jk+11−1−ϑiLp1−ϑjLq,∏i,j=1i≠jk+11−1−ϑiUp1−ϑjUq]〉,∏i,j=1i≠jk+11−1−ζip1−ζjq,1−∏i,j=1i≠jk+11−(ϑi)p(ϑj)q

which is true for n=k+1 and hence by principle of mathematical induction, Equation (16) holds for all positive integers *n*.

Now,

(22) 1n(n−1)⨁i,j=1i≠jnδip⊗δjq=〈[1−∏i,j=1i≠jn1−(ζiL)p(ζjL)q1n(n−1),1−∏i,j=1i≠jn1−(ζiU)p(ζjU)q1n(n−1)],[∏i,j=1i≠jn1−1−ϑiLp1−ϑjLq1n(n−1),∏i,j=1i≠jn1−1−ϑiUp1−ϑjUq1n(n−1)]〉,∏i,j=1i≠jn1−1−ζip1−ζjq1n(n−1),1−∏i,j=1i≠jn(1−(ϑi)p(ϑj)q)1n(n−1)

So by definition of CIFBM, we get

(23) CIFBMp,q(δ1,δ2,…,δn)=1n(n−1)⨁i,j=1i≠jnδip⊗δjq1p+q=〈1−∏i,j=1i≠jn1−(ζiL)p(ζjL)q1n(n−1)1p+q,1−∏i,j=1i≠jn1−(ζiU)p(ζjU)q1n(n−1)1p+q,1−1−∏i,j=1i≠jn1−(1−ϑiL)p(1−ϑjL)q1n(n−1)1p+q,1−1−∏i,j=1i≠jn1−(1−ϑiU)p(1−ϑjU)q1n(n−1)1p+q〉,1−1−∏i,j=1i≠jn1−(1−ζi)p(1−ζj)q1n(n−1)1p+q,1−∏i,j=1i≠jn1−(ϑi)p(ϑj)q1n(n−1)1p+q

Hence, the result.

Finally, in order to show the aggregated value by using CIFBM is also CIFN, it is necessary to satisfy the CIFN property. For it, since $\delta_{i}=\left(\langle \left[\zeta_{i}^{L},\zeta_{i}^{U}\right],\left[\vartheta_{i}^{L},\vartheta_{i}^{U}\right]\rangle ,\langle \zeta_{i},\vartheta_{i}\rangle \right)$ is CIFN which implies that $\left[\zeta_{i}^{L},\zeta_{i}^{U}\right],\left[\vartheta_{i}^{L},\vartheta_{i}^{U}\right]\subseteq \left[0,1\right]$ and $\zeta_{i}^{U}+\vartheta_{i}^{U}\leq 1$, $\zeta_{i},\vartheta_{i}B\left[0,1\right]$ and $\zeta_{i}+\vartheta_{i}\leq 1$. Thus, for any positive number *p* and *q*, we have $0\leq 1-{\left(\zeta_{i}^{U}\right)}^{p}{\left(\zeta_{j}^{U}\right)}^{q}\leq 1$ which turns $0\leq {\left(1-{\left(\zeta_{i}^{U}\right)}^{p}{\left(\zeta_{j}^{U}\right)}^{q}\right)}^{\frac{1}{n(n-1)}}\leq 1$ and hence 0≤1−∏i,j=1i≠jn1−(ζiU)p(ζjU)q1n(n−1)1p+q≤1. On the other hand, $0\leq \vartheta_{i}^{U},\vartheta_{j}^{U}\leq 1$, thus $0\leq {\left(1-\vartheta_{i}^{U}\right)}^{p}{\left(1-\vartheta_{j}^{U}\right)}^{q}\leq 1$, which further gives 0≤1−1−∏i,j=1i≠jn1−(1−ϑiU)p(1−ϑjU)q1n(n−1)1p+q≤1. Lastly, from $\zeta_{i}^{U}+\vartheta_{i}^{U}\leq 1$, we have ${\left(\zeta_{i}^{U}\right)}^{p}\leq {\left(1-\vartheta_{i}^{U}\right)}^{p}$ and ${\left(\zeta_{j}^{U}\right)}^{q}\leq {\left(1-\vartheta_{j}^{U}\right)}^{q}$ and thus follows that ∏i,j=1i≠jn1−(ζiU)p(ζjU)q1n(n−1)≥∏i,j=1i≠jn1−(1−ϑiU)p(1−ϑjU)q1n(n−1) which in turns leads us to

1−∏i,j=1i≠jn1−(ζiU)p(ζjU)q1n(n−1)1p+q+1−1−∏i,j=1i≠jn1−(1−ϑiU)p(1−ϑjU)q1n(n−1)1p+q=1−1−∏i,j=1i≠jn1−(1−ϑiU)p(1−ϑjU)q1n(n−1)1p+q−1−∏i,j=1i≠jn1−(ζiU)p(ζjU)q1n(n−1)1p+q≤1

Similarly, we can prove for remaining components of CIFBM and hence the aggregated value by CIFBM operator is again CIFN. This completes the proof. ☐

From CIFBM operator, it is observed that they satisfies certain properties for a collection of CIFN $\delta_{i}$, which are stated as follows:

**Property** **1.** *(Idempotency) If $\delta_{i}=\delta$ for all i, then CIFBM satsifies*

$$\begin{array}{c} {\mathrm{CIFBM}}^{p,q}\left(\delta ,\delta ,\ldots \delta \right)=\delta \end{array}$$

**Proof.**  Assume $\delta =\left(\langle \left[\zeta^{L},\zeta^{U}\right],\left[\vartheta^{L},\vartheta^{U}\right]\rangle ,\langle \zeta ,\vartheta \rangle \right)$ and $\delta_{i}=\delta$ for all *i*, then we have

CIFBMp,q(δ,δ,…δ)=〈[(1−∏i,j=1i≠jn1−(ζL)p(ζL)q1n(n−1))1p+q,1−∏i,j=1i≠jn1−(ζU)p(ζU)q1n(n−1)1p+q],[1−(1−∏i,j=1i≠jn1−(1−ϑL)p(1−ϑL)q1n(n−1))1p+q,1−(1−∏i,j=1i≠jn1−(1−ϑU)p(1−ϑU)q1n(n−1))1p+q]〉,1−(1−∏i,j=1i≠jn1−(1−ζ)p(1−ζ)q1n(n−1))1p+q,1−∏i,j=1i≠jn1−(ϑ)p(ϑ)q1n(n−1)1p+q=〈1−1−(ζL)p+q1p+q,1−1−(ζU)p+q1p+q,[1−1−1−(1−(ϑL)p+q)1p+q,1−1−1−(1−(ϑU)p+q)1p+q]〉,1−1−1−(1−(ζ)p+q)1p+q,1−1−(ϑ)p+q1p+q=〈(ζL)p+q1p+q,(ζU)p+q1p+q,[1−(1−ϑL)p+q1p+q,1−(1−ϑU)p+q1p+q]〉,1−(1−ζ)p+q1p+q,(ϑ)p+q1p+q=[ζL,ζU],[ϑL,ϑU],ζ,ϑ=δ

 ☐

**Property** **2.** *(Monotonicity) Let $\delta_{i}=\left(\langle \left[\zeta_{\delta_{i}}^{L},\zeta_{\delta_{i}}^{U}\right],\left[\vartheta_{\delta_{i}}^{L},\vartheta_{\delta_{i}}^{U}\right]\rangle ,\langle \zeta_{\delta_{i}},\vartheta_{\delta_{i}}\rangle \right)$ and $\beta_{i}=\left(\langle \left[\zeta_{\beta_{i}}^{L},\zeta_{\beta_{i}}^{U}\right],\left[\vartheta_{\beta_{i}}^{L},\vartheta_{\beta_{i}}^{U}\right]\rangle ,\langle \zeta_{\beta_{i}},\vartheta_{\beta_{i}}\rangle \right)$ be any two CIFNs such that $\zeta_{\delta_{i}}^{L}\leq \zeta_{\beta_{i}}^{L}$, $\zeta_{\delta_{i}}^{U}\leq \zeta_{\delta_{i}}^{U}$, $\vartheta_{\delta_{i}}^{L}\geq \vartheta_{\beta_{i}}^{U}$, $\vartheta_{\delta_{i}}^{U}\geq \vartheta_{\beta_{i}}^{U}$ and $\zeta_{\delta_{i}}\geq \zeta_{\beta_{i}}$, $\vartheta_{\delta_{i}}\leq \vartheta_{\beta_{i}}$, then*

$${\mathrm{CIFBM}}^{p,q}\left(\delta_{1},\delta_{2},\ldots ,\delta_{n}\right)\leq {\mathrm{CIFBM}}^{p,q}\left(\beta_{1},\beta_{2},\ldots ,\beta_{n}\right)$$

*.*

**Proof.**  Let ${\mathrm{CIFBM}}^{p,q}\left(\delta_{1},\delta_{2},\ldots ,\delta_{n}\right)=\left(\langle \left[\zeta_{\delta }^{L},\zeta_{\delta }^{U}\right],\left[\vartheta_{\delta }^{L},\vartheta_{\delta }^{U}\right]\rangle ,\langle \zeta_{\delta },\vartheta_{\delta }\rangle \right)$ and ${\mathrm{CIFBM}}^{p,q}\left(\beta_{1},\beta_{2},\ldots ,\beta_{n}\right)=\left(\langle \left[\zeta_{\beta }^{L},\zeta_{\beta }^{U}\right]\right.$, $\left.\left[\vartheta_{\beta }^{L},\vartheta_{\beta }^{U}\right]\rangle ,\langle \zeta_{\beta },\vartheta_{\beta }\rangle \right)$. Now, for any two CIFNs $\delta_{i}$ and $\delta_{j}$, and by using relation that $\zeta_{\delta_{i}}^{U}\leq \zeta_{\delta_{i}}^{U}$, we have

ζδiUpζδjUq≤ζβiUpζβjUq⇔∏i,j=1i≠jn1−ζβiUpζβjUq1n(n−1)≤∏i,j=1i≠jn1−ζδiUpζδjUq1n(n−1)⇔1−∏i,j=1i≠jn1−ζδiUpζδjUq1n(n−1)≤1−∏i,j=1i≠jn1−ζβiUpζβjUq1n(n−1)⇔1−∏i,j=1i≠jn1−ζδiUpζδjUq1n(n−1)1p+q≤1−∏i,j=1i≠jn1−ζβiUpζβjUq1n(n−1)1p+qi.e.,ζδU≤ζβU

Similarly,

1−∏i,j=1i≠jn1−ζδiLpζδjLq1n(n−1)1p+q≤1−∏i,j=1i≠jn1−ζβiLpζβjLq1n(n−1)1p+qand1−∏i,j=1i≠jn1−ϑδipϑδjq1n(n−1)1p+q≤1−∏i,j=1i≠jn1−ϑβipϑβjq1n(n−1)1p+q

On the other hand, for $\vartheta_{\delta_{i}}^{U}\leq \vartheta_{\beta_{i}}^{U}$ and hence for any two CIFNs $\delta_{i}$ and $\delta_{j}$, we have

1−ϑδiUp1−ϑδjUq≤1−ϑβiUp1−ϑβjUq⇔∏i,j=1i≠jn1−1−ϑβiUp1−ϑβjUq1n(n−1)≤∏i,j=1i≠jn1−1−ϑδiUp1−ϑδjUq1n(n−1)⇔1−∏i,j=1i≠jn1−1−ϑδiUp1−ϑδjUq1n(n−1)1p+q≤1−∏i,j=1i≠jn1−1−ϑβiUp1−ϑβjUq1n(n−1)1p+q⇔1−1−∏i,j=1i≠jn1−1−ϑβiUp1−ϑβjUq1n(n−1)1p+q≤1−1−∏i,j=1i≠jn1−1−ϑδiUp1−ϑδjUq1n(n−1)1p+q

Similarly,

1−1−∏i,j=1i≠jn1−1−ϑβiLp1−ϑβjLq1n(n−1)1p+q≤1−1−∏i,j=1i≠jn1−1−ϑδiLp1−ϑδjLq1n(n−1)1p+qand1−1−∏i,j=1i≠jn1−1−ζβip1−ζβjq1n(n−1)1p+q≤1−1−∏i,j=1i≠jn1−1−ζδip1−ζδjq1n(n−1)1p+q

Thus,

$$\begin{array}{ccc} \mathrm{Sc}({\mathrm{CIFBM}}^{p,q}\left(\delta_{1},\delta_{2},\ldots ,\delta_{n}\right)) & = & \frac{\zeta_{\delta }^{L}+\zeta_{\delta }^{U}-\vartheta_{\delta }^{L}-\vartheta_{\delta }^{U}}{2}-\zeta_{\delta }+\vartheta_{\delta }\\ & \leq & \frac{\zeta_{\beta }^{L}+\zeta_{\beta }^{U}-\vartheta_{\beta }^{L}-\vartheta_{\beta }^{U}}{2}-\zeta_{\beta }+\vartheta_{\beta }\\ & = & \mathrm{Sc}({\mathrm{CIFBM}}^{p,q}\left(\beta_{1},\beta_{2},\ldots ,\beta_{n}\right)) \end{array}$$

Hence, by comparison law, we have ${\mathrm{CIFBM}}^{p,q}\left(\delta_{1},\delta_{2},\ldots ,\delta_{n}\right)\leq {\mathrm{CIFBM}}^{p,q}\left(\beta_{1},\beta_{2},\ldots ,\beta_{n}\right)$. ☐

**Property** **3.** (Commutativity) If $\left(\dot{\delta_{1}},\dot{\delta_{2}},\ldots ,\dot{\delta_{n}}\right)$ be any permutation of CIFNs $\left(\delta_{1},\delta_{2},\ldots ,\delta_{n}\right)$, then ${\mathrm{CIFBM}}^{p,q}\left(\delta_{1},\delta_{2},\ldots ,\delta_{n}\right)={\mathrm{CIFBM}}^{p,q}\left(\dot{\delta_{1}},\dot{\delta_{2}},\ldots ,\dot{\delta_{n}}\right)$.

**Proof.**  For permutation $\left(\dot{\delta_{1}},\dot{\delta_{2}},\ldots ,\dot{\delta_{n}}\right)$ of $\left(\delta_{1},\delta_{2},\ldots ,\delta_{n}\right)$,

CIFBMp,q(δ1,δ2,…,δn)=1n(n−1)⨁i,j=1i≠jnδip⊗δjq1p+q=1n(n−1)⨁i,j=1i≠jnδ˙ip⊗δ˙jq1p+q=CIFBMp,q(δ1˙,δ2˙,…,δn˙)

 ☐

**Property** **4.** *(Boundedness) Let $\delta^{-}=\langle \left[\zeta_{\min}^{L},\zeta_{\min}^{U}\right],\left[\vartheta_{\max}^{L},\vartheta_{\max}^{U}\right]\rangle ,\langle \zeta_{\max},\vartheta_{\min}\rangle \rangle$, $\delta^{+}=\langle \left[\zeta_{\max}^{L},\zeta_{\max}^{U}\right]$, $\left[\vartheta_{\min}^{L},\vartheta_{\min}^{U}\right]\rangle ,\langle \zeta_{\min},\vartheta_{\max}\rangle \rangle$ are the lower and upper bounds for the collection of CIFNs $\delta_{i}$, then*

$$\begin{array}{c} \delta^{-}\leq {\mathrm{CIFBM}}^{p,q}\left(\delta_{1},\ldots ,\delta_{n}\right)\leq \delta^{+}. \end{array}$$

**Proof.**  Since, $\zeta_{\min}^{L}\leq \zeta_{i}^{L}\leq \zeta_{\max}^{L}$ and $\zeta_{\min}^{U}\leq \zeta_{i}^{U}\leq \zeta_{\max}^{U}$ which implies that

ζminLp+q≤(ζiU)p(ζjL)q≤ζmaxLp+q⇔∏i,j=1i≠jn1−ζmaxLp+q1n(n−1)≤∏i,j=1i≠jn1−ζiLpζjLq1n(n−1)≤∏i,j=1i≠jn1−ζminLp+q1n(n−1)⇔1−ζmaxLp+q≤∏i,j=1i≠jn1−ζiLpζjLq1n(n−1)≤1−ζminLp+q⇔ζminLp+q≤1−∏i,j=1i≠jn1−ζiLpζjLq1n(n−1)≤ζmaxLp+q⇔ζminL≤1−∏i,j=1i≠jn1−ζiLpζjLq1n(n−1)1p+q≤ζmaxL

Similarly, we get

ζminU≤1−∏i,j=1i≠jn1−ζiUpζjUq1n(n−1)1p+q≤ζmaxUandϑmin≤1−∏i,j=1i≠jn1−ϑipϑjq1n(n−1)1p+q≤ϑmax

On the other hand, for $\vartheta_{\min}^{L}\leq \vartheta_{i}^{L}\leq \vartheta_{\max}^{L}$ and $\vartheta_{\min}^{U}\leq \vartheta_{i}^{U}\leq \vartheta_{\max}^{U}$, we have

1−ϑmaxUp+q≤1−ϑiUp1−ϑjUq≤1−ϑminUp+q⇔1−1−ϑminUp+q≤1−1−ϑiUp1−ϑjUq≤1−1−ϑmaxUp+q⇔1−1−ϑminUp+q≤∏i,j=1i≠jn1−1−ϑiUp1−ϑjUq1n(n−1)≤1−1−ϑmaxUp+q⇔1−ϑmaxUp+q≤1−∏i,j=1i≠jn1−1−ϑiUp1−ϑjUq1n(n−1)≤1−ϑminUp+q⇔1−ϑmaxU≤1−∏i,j=1i≠jn1−1−ϑiUp1−ϑjUq1n(n−1)1p+q≤1−ϑminU⇔ϑminU≤1−1−∏i,j=1i≠jn1−1−ϑiUp1−ϑjUq1n(n−1)1p+q≤ϑmaxU

Similarly, we have

ϑminL≤1−1−∏i,j=1i≠jn1−1−ϑiLp1−ϑjLq1n(n−1)1p+q≤ϑmaxLandζmin≤1−1−∏i,j=1i≠jn1−1−ζip1−ζjq1n(n−1)1p+q≤ζmax

Thus, by comparing the two CIFNs, we get $\delta^{-}\leq {\mathrm{CIFBM}}^{p,q}\left(\delta_{1},\delta_{2},\ldots ,\delta_{n}\right)\leq \delta^{+}$. ☐

In the following, we will discuss some special cases of CIFBM operator by taking different values of *p* and *q*.

(Case 1) As q→0, then Equation (7) reduces to generalized cubic intuitionistic fuzzy mean which is defined as follows:
  $$\begin{array}{cc} & {\mathrm{CIFBM}}^{p,q}\left(\delta_{1},\delta_{2},\ldots ,\delta_{n}\right)\\ = & \left(\begin{array}{c} \langle \left[{\left(1-\prod_{i=1}^{n}{\left(1-{\left(\zeta_{i}^{L}\right)}^{p}\right)}^{\frac{n-1}{n(n-1)}}\right)}^{\frac{1}{p}},{\left(1-\prod_{i=1}^{n}{\left(1-{\left(\zeta_{i}^{U}\right)}^{p}\right)}^{\frac{n-1}{n(n-1)}}\right)}^{\frac{1}{p}}\right],\\ \left[1-{\left(1-\prod_{i=1}^{n}{\left(1-{\left(1-\vartheta_{i}^{L}\right)}^{p}\right)}^{\frac{n-1}{n(n-1)}}\right)}^{\frac{1}{p}},1-{\left(1-\prod_{i=1}^{n}{\left(1-{\left(1-\vartheta_{i}^{U}\right)}^{p}\right)}^{\frac{n-1}{n(n-1)}}\right)}^{\frac{1}{p}}\right]\rangle ,\\ \langle 1-{\left(1-\prod_{i=1}^{n}{\left(1-{\left(1-\zeta_{i}\right)}^{p}\right)}^{\frac{n-1}{n(n-1)}}\right)}^{\frac{1}{p}},{\left(1-\prod_{i=1}^{n}{\left(1-{\left(\vartheta_{i}\right)}^{p}\right)}^{\frac{n-1}{n(n-1)}}\right)}^{\frac{1}{p}}\rangle \end{array}\right)\\ = & \left(\begin{array}{c} \langle \left[{\left(1-\prod_{i=1}^{n}{\left(1-{\left(\zeta_{i}^{L}\right)}^{p}\right)}^{\frac{1}{n}}\right)}^{\frac{1}{p}},{\left(1-\prod_{i=1}^{n}{\left(1-{\left(\zeta_{i}^{U}\right)}^{p}\right)}^{\frac{1}{n}}\right)}^{\frac{1}{p}}\right],\\ \left[1-{\left(1-\prod_{i=1}^{n}{\left(1-{\left(1-\vartheta_{i}^{L}\right)}^{p}\right)}^{\frac{1}{n}}\right)}^{\frac{1}{p}},1-{\left(1-\prod_{i=1}^{n}{\left(1-{\left(1-\vartheta_{i}^{U}\right)}^{p}\right)}^{\frac{1}{n}}\right)}^{\frac{1}{p}}\right]\rangle ,\\ \langle 1-{\left(1-\prod_{i=1}^{n}{\left(1-{\left(1-\zeta_{i}\right)}^{p}\right)}^{\frac{1}{n}}\right)}^{\frac{1}{p}},{\left(1-\prod_{i=1}^{n}{\left(1-{\left(\vartheta_{i}\right)}^{p}\right)}^{\frac{1}{n}}\right)}^{\frac{1}{p}}\rangle \end{array}\right)\\ = & {\left(\frac{1}{n}\left(\overset{n}{\underset{i=1}{⨁}}\delta_{i}^{p}\right)\right)}^{\frac{1}{p}}\\ = & {\mathrm{CIFBM}}^{p,0}\left(\delta_{1},\delta_{2},\ldots ,\delta_{n}\right) \end{array}$$
(Case 2) If p=2 and as q→0, then Equation (7) reduces to cubic intuitionistic fuzzy square mean which is given as follows:
  $$\begin{array}{cc} & {\mathrm{CIFBM}}^{p,q}\left(\delta_{1},\delta_{2},\ldots ,\delta_{n}\right)\\ = & \left(\begin{array}{c} \langle [{\left(1-{\left(\prod_{i=1}^{n}\left(1-{\left(\zeta_{i}^{L}\right)}^{2}\right)\right)}^{\frac{1}{n}}\right)}^{\frac{1}{2}},\left(1-{\left(\prod_{i=1}^{n}{\left(1-{\left(\zeta_{i}^{U}\right)}^{2})\right)}^{\frac{1}{n}}\right)}^{\frac{1}{2}}\right],\\ \left[1-{\left(1-{\left(\prod_{i=1}^{n}\left(1-{\left(1-\vartheta_{i}^{L}\right)}^{2}\right)\right)}^{\frac{1}{n}}\right)}^{\frac{1}{2}},1-{\left(1-{\left(\prod_{i=1}^{n}\left(1-{\left(1-\vartheta_{i}^{U}\right)}^{2}\right)\right)}^{\frac{1}{n}}\right)}^{\frac{1}{2}}\right]\rangle ,\\ \langle 1-{\left(1-{\left(\prod_{i=1}^{n}\left(1-{\left(1-\zeta_{i}\right)}^{2}\right)\right)}^{\frac{1}{n}}\right)}^{\frac{1}{2}},{\left(1-{\left(\prod_{i=1}^{n}\left(1-{\left(\vartheta_{i}\right)}^{2}\right)\right)}^{\frac{1}{n}}\right)}^{\frac{1}{2}}\rangle \end{array}\right) \end{array}$$
  $$\begin{array}{c} ={\left(\frac{1}{n}\overset{n}{\underset{i=1}{⨁}}\delta_{i}^{2}\right)}^{\frac{1}{2}} \end{array}$$
(Case 3) For p=1 and q→0, Equation (7) becomes cubic intuitionistic fuzzy average operator as:
  $$\begin{array}{cc} & {\mathrm{CIFBM}}^{1,0}\left(\delta_{1},\delta_{2},\ldots ,\delta_{n}\right)\\ = & \left(\begin{array}{c} \langle \left[\left(1-{\left(\prod_{i=1}^{n}\left(1-\left(\zeta_{i}^{L}\right)\right)\right)}^{\frac{1}{n}}\right),{\left(1-\left(\prod_{i=1}^{n}\left(1-\left(\zeta_{i}^{U}\right)\right)\right)\right)}^{\frac{1}{2}}\right],\\ \left[1-\left(1-{\left(\prod_{i=1}^{n}\left(1-\left(1-\vartheta_{i}^{L}\right)\right)\right)}^{\frac{1}{n}}\right),1-\left(1-{\left(\prod_{i=1}^{n}\left(1-\left(1-\vartheta_{i}^{U}\right)\right)\right)}^{\frac{1}{n}}\right)\right]\rangle ,\\ \langle 1-\left(1-{\left(\prod_{i=1}^{n}\left(1-\left(1-\zeta_{i}\right)\right)\right)}^{\frac{1}{n}}\right),\left(1-{\left(\prod_{i=1}^{n}\left(1-\left(\vartheta_{i}\right)\right)\right)}^{\frac{1}{n}}\right)\rangle \end{array}\right)\\ = & \frac{1}{n}\overset{n}{\underset{i=1}{⨁}}\delta_{i} \end{array}$$
(Case 4) For p=q=1, Equation (7) reduces to cubic intuitionistic interrelated square mean which is defined as
  CIFBM1,1(δ1,δ2,…,δn)=〈1n(n−1)⨁i,j=1i≠jn(1−ζiLζjL)1n(n−1)12,1n(n−1)⨁i,j=1i≠jn(1−ζiUζjU)1n(n−1)12,[1−1−∏i,j=1i≠j(1−(1−ϑiL)(1−ϑiL))1n(n−1)12,1−1−∏i,j=1i≠j(1−(1−ϑiU)(1−ϑiU))1n(n−1)12]〉,1−1−∏i,j=1i≠j(1−(1−ζi)(1−ζi))1n(n−1)12,1n(n−1)⨁i,j=1i≠jn(1−ϑiϑj)1n(n−1)12

### 3.3. Weighted BM Operator of CIFNs

**Definition** **17.** *For CIFNs $\delta_{i}$(i=1,2,…,n) and weight vector $\kappa ={\left(\kappa_{1},\kappa_{2},\ldots ,\kappa_{n}\right)}^{T}$ such that each $\kappa_{i}>0$ and $\sum_{i=1}^{n}\kappa_{i}=1$, a weighted CIFBM defined over family of CIFNs* Ω *as WCIFBM : $\Omega^{n}\to \Omega$ and is given by*

(24) WCIFBMκp,q(δ1,δ2,…,δn)=1n(n−1)⨁i,j=1i≠jnκiδip⊗κjδjq1p+q

*for a positive real number p ad q.*

**Theorem** **4.** *The aggregated value by using WCIFBM operator for collection of CIFNs $\delta_{i}=(\langle \left[\zeta_{i}^{L},\zeta_{i}^{U}\right]$, $[\vartheta_{i}^{L},\vartheta_{i}^{U}]\rangle$, $\langle \zeta_{i},\vartheta_{i}\rangle )$, i=(1,2,…,n) is also CIFN and can be expressed as*

(25) $$\begin{array}{c} {\mathrm{WCIFBM}}_{\kappa }^{p,q}\left(\delta_{1},\delta_{2},\ldots ,\delta_{n}\right)=\left(\langle \left[\zeta^{L},\zeta^{U}\right],\left[\vartheta^{L},\vartheta^{U}\right]\rangle ,\langle \zeta ,\vartheta \rangle \right) \end{array}$$

*where*

ζL=1−∏i,j=1i≠jn1−1−1−ζiLκip1−1−ζjLκjq1n(n−1)1p+qζU=1−∏i,j=1i≠jn1−1−1−ζiUκip1−1−ζjUκjq1n(n−1)1p+qϑL=1−1−∏i,j=1i≠jn1−1−ϑiLκip1−ϑjLκjq1n(n−1)1p+qϑU=1−1−∏i,j=1i≠jn1−1−ϑiUκip1−ϑjUκjq1n(n−1)1p+qζ=1−1−∏i,j=1i≠jn1−1−ζiκip1−ζjκjq1n(n−1)1p+qϑ=1−∏i,j=1i≠jn1−1−1−ϑiκip1−1−ϑjκjq1n(n−1)1p+q

*and $\kappa ={\left(\kappa_{1},\kappa_{2},\ldots ,\kappa_{n}\right)}^{T}$ be the associated weight vector such that each $\kappa_{i}>0$ and $\sum_{i=1}^{n}\kappa_{i}=1$.*

**Proof.**  Proof is similar to that of Theorem 3, so we omit here. ☐

## 4. Proposed Decision-Making Approach Based of Cubic Intuitionistic Fuzzy Bonferroni Mean Operator

In this section, we shall utilize the proposed Bonferroni mean aggregation operator to solve the multi-attribute decision making under the cubic intuitionistic fuzzy sets environment. For it, the following assumptions or notations are used to present the MADM problems for evaluating these with a cubic intuitionistic fuzzy set environment. Let $A=\{A_{1},A_{2},\ldots ,A_{m}\}$ be the set of *m* different alternatives which have to be analyzed under the set of ‘*n*’ different criteria $C=\{C_{1},C_{2},\ldots ,C_{n}\}$. Assume that these alternatives are evaluated by an expert which give their preferences related to each alternative $A_{i}\left(i=1,2,\ldots ,m\right)$ under the CIFSs environment, and these values can be considered as CIFNs $D={\left(\delta_{ij}\right)}_{m\times n}$ where $\delta_{ij}=(\langle \left[\zeta_{ij}^{L},\zeta_{ij}^{U}\right]$, $[\vartheta_{ij}^{L},\vartheta_{ij}^{U}]\rangle$, $\langle \zeta_{ij},\vartheta_{ij}\rangle )$ represents the priority values of alternative $A_{i}$ given by decision maker such that $\left[\zeta_{ij}^{L},\zeta_{ij}^{U}\right],\left[\vartheta_{ij}^{L},\vartheta_{ij}^{U}\right]\subseteq \left[0,1\right]$, $\zeta_{ij},\vartheta_{ij}\in \left[0,1\right]$ and $\zeta_{ij}^{U}+\vartheta_{ij}^{U}\leq 1$, $\zeta_{ij}+\vartheta_{ij}\leq 1$ for i=1,2,…,m;j=1,2,…,n. Let $\kappa ={\left(\kappa_{1},\kappa_{2},\ldots ,\kappa_{n}\right)}^{T}$ be the weight vector of the criteria such that $\kappa_{j}>0$ and $\sum_{j=1}^{n}\kappa_{j}=1$. Then, the proposed method has been summarized into the various steps which are described as follows to find the best alternative(s).

Step 1: Collect the information rating of alternatives corresponding to criteria and summarize in the form of CIFN $\delta_{ij}=(\langle \left[\zeta_{ij}^{L},\zeta_{ij}^{U}\right]$, $[\vartheta_{ij}^{L},\vartheta_{ij}^{U}]\rangle$, $\langle \zeta_{ij},\vartheta_{ij}\rangle )$ : i=1,2,…,m; j=1,2,…,n. These rating values are expressed as a decision matrix *D* as
  $$D=\begin{array}{cc} & \begin{array}{cccc} C_{1} & C_{2} & \ldots & C_{n} \end{array}\\ \begin{array}{c} A_{1}\\ A_{2}\\ \vdots \\ A_{n} \end{array} & \left(\begin{array}{cccc} \delta_{11} & \delta_{12} & \ldots & \delta_{1n}\\ \delta_{21} & \delta_{22} & \ldots & \delta_{2n}\\ \vdots & \vdots & \ddots & \\ \delta_{m1} & \delta_{m2} & \ldots & \delta_{mn} \end{array}\right) \end{array}$$
Step 2: Normalize these collective information decision matrix by transforming the rating values of cost type into benefit type, if any, by using the normalization formula:
  (26) $$\begin{array}{c} r_{ij}=\left\{\begin{array}{cc} \left(\langle \left[\zeta_{ij}^{L},\zeta_{ij}^{U}\right],\left[\vartheta_{ij}^{L},\vartheta_{ij}^{U}\right]\rangle ,\langle \zeta_{ij},\vartheta_{ij}\rangle \right) & ;\;\mathrm{for}\;\mathrm{benefit}\;\mathrm{type}\;\mathrm{criterion}\\ \left(\langle \left[\vartheta_{ij}^{L},\vartheta_{ij}^{U}\right],\left[\zeta_{ij}^{L},\zeta_{ij}^{U}\right]\rangle ,\langle \vartheta_{ij},\zeta_{ij}\rangle \right) & ;\;\mathrm{for}\;\cos t\;\mathrm{type}\;\mathrm{criterion} \end{array}\right. \end{array}$$
  and hence summarize it into the decision matrix $R={\left(r_{ij}\right)}_{m\times n}$.
Step 3: Aggregate the different preference values $r_{ij},j=1,2,\ldots ,n$ of the alternatives $A_{i}$ into the collective one $r_{i},i=1,2,\ldots ,m$ by using WCIFBM aggregation operator for a real positive number p,q as
  $$\begin{array}{ccc} r_{i} & = & \left(\langle \left[\zeta_{ij}^{L},\zeta_{ij}^{U}\right],\left[\vartheta_{ij}^{L},\vartheta_{ij}^{U}\right]\rangle ,\langle [\zeta_{ij},\vartheta_{ij}]\rangle \right)\\ & = & {\mathrm{WCIFBM}}^{p,q}\left(r_{i1},r_{i2},\ldots ,r_{in}\right) \end{array}$$
Step 4: Compute the score value of the aggregated CIFN $r_{i}$ by using Equation (4) as
  (27) $$\begin{array}{c} \mathrm{Sc}\left(r_{i}\right)=\frac{\zeta_{i}^{L}+\zeta_{i}^{U}-\vartheta_{i}^{L}-\vartheta_{i}^{U}}{2}+\left(\vartheta_{i}-\zeta_{i}\right) \end{array}$$
Step 5: Rank the alternative $A_{i},i=1,2,\ldots ,m$ with the order of their score value $\mathrm{Sc}(r_{i})$.

## 5. Illustrative Example

For demonstrating the real-life application of the proposed approach, a numerical example has been illustrated below:

### 5.1. Case Study

Inventory management is an issue of great concern these days. From an industrial viewpoint, a company cannot excel in desired levels of manufacturing until its inventory is not managed properly. Therefore, proper inventory management is the first step of the ladder of good production levels. Any shortage of raw material in inventory may disrupt the whole manufacturing cycle which in-turn can incur a huge loss to the company. Suppose, a Food company wants to keep track of various inventory items. The company produces mainly four kinds of food $\left(A_{i}\right)$’s namely “Beverages”, “Edible oils”, “Pickles” and “Bakery items”. For manufacturing these food items, the stock re-ordering decisions for ingredients in inventory are to be taken on account of three factors $C_{j}$’s as “Cost Price”, “Storage facilities” and “Staleness level”. The weight vector of these factors is taken as $\kappa ={\left(0.20,0.38,0.42\right)}^{T}$. The given alternatives are evaluated under these three factors and rate their values in terms of CIFNs. In each CIFN, the IVIFNs shows the existing stock level in the inventory and the IFNs represent the estimate of agreeness as well as disagreeness towards the present stock level for a coming week. Since the company does not compromise with the quality of production, therefore maximum priority is given to reduce staleness levels. Then, the aim is to identify the food-items whose ingredients’ stock is needed to be re-ordered frequently. For it, the following steps of the proposed approach have been executed as follows.

Step 1: The preferences information related to each alternative are summarized in CIFNs and the collection rating are given in the decision matrix as shown in Table 1.
Step 2: By using Equation (26), we obtain normalized CIFNs and summarized in Table 2.
Step 3: For the sake of simplicity, we choose p=q=1 and then by using Equation (25) to compute the overall value of each alternative as $r_{1}=(\langle \left[0.0601,0.0988\right]$, [0.7589,0.8305]〉, 〈0.6681, 0.0892〉), $r_{2}=(\langle \left[0.0648,0.1069\right]$, [0.6265,0.7146]〉,〈0.7503,0.1361〉), $r_{3}=(\langle [0.0758$, 0.1215], [0.7480, 0.8254]〉, 〈0.7436, 0.1132〉) and $r_{4}=(\langle \left[0.0844,0.1463\right]$,[0.6609,0.7358]〉, 〈0.6908, 0.1264〉).
Step 4: By using Equation (4), the score value of each alternative is obtained as $\mathrm{Sc}(r_{1})=-1.2942$, $\mathrm{Sc}(r_{2})=-1.1989$, $\mathrm{Sc}(r_{3})=-1.3185$ and $\mathrm{Sc}(r_{4})=-1.1474$.
Step 5: The ranking order of the alternatives based on the score values is found to be $A_{4}≻A_{2}≻A_{1}≻A_{3}$. Thus, Bakery items’ stock needs maximum re-ordering.

The proposed aggregation operators are symmetric with respect to the parameters *p* and *q*. However, in order to analyze the effect of these parameters on to the final ranking of the alternatives, an investigation has been done by varying it simultaneously and their score values along with ranking order are summarized in Table 3. From this table, we can find that by assigning different pairs of the parameters *p* and *q*, the score values of the aggregated numbers are different; however, the ranking orders of the alternatives remain same. This feature of the proposed operators is more crucial in real decision-making problems. For instance, it has been seen that with the increase of the parameters, the score values of the alternative increases, which gives us optimism view to the decision makers’. Therefore, if the decision makers are optimistic then the higher values can be assigned to these parameters during the aggregation process. On the other hand, if the decision makers are pessimistic then lower values can be assigned to these parameters and the score values of the overall values are decreasing. However, the best alternative is the same, which influenced that the results are objective and cannot be changed by decision makes’ preference of pessimism and optimism. Thus, the ranking results are reliable.

**Figure 1:**
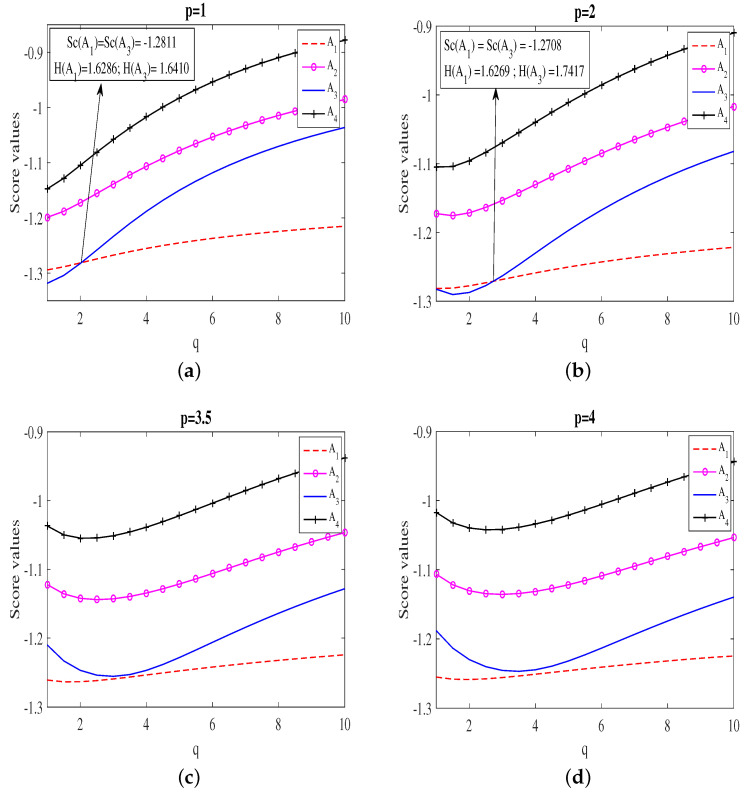
Effect of the parameter *q* on to the score value by fixing the parameter *p*.

On the other hand, the variations of the complete score values of each alternative by varying one of the parameter *p* are summarized in Figure 1. It can be analyzed from Figure 1, that the maximum score possessing alternative remains $A_{4}$ for all cases. However, in Figure 1a, by fixing the parameter p=1, and varying *q* from 0 to 10, it is observed that when q<2.0307, alternative $A_{3}$ shows least scores whereas for q>2.0307, $A_{1}$ possesses least score values. However, at q=2.0307, Sc$\left(A_{1}\right)$ = Sc$\left(A_{3}\right)$ = −1.2811 and thus, from the accuracy function, we get $H(A_{1})=1.6286$ and $H(A_{3})=1.6410.$ which implies that the ranking order of the alternatives at p=1 and $q=q^{\prime }=2.0307$ is given as $A_{4}≻A_{2}≻A_{3}≻A_{1}.$ Therefore, the worst alternative changes from $A_{3}$ to $A_{1}$. Similarly, in Figure 1b, we observed that when q<2.742, the worst alternative is $A_{3}$ white it is $A_{1}$ when q>2.742 corresponding to p=2. Further, $q=q^{\prime }=2.742$, the ranking order of the alternatives is $A_{4}≻A_{2}≻A_{3}≻A_{1}.$ The complete rating values for all the alternatives are summarized in Table 4.

### 5.2. Graphical Analysis of Obtained Score Values Based on WCIFBM Operator

**Figure 2:**
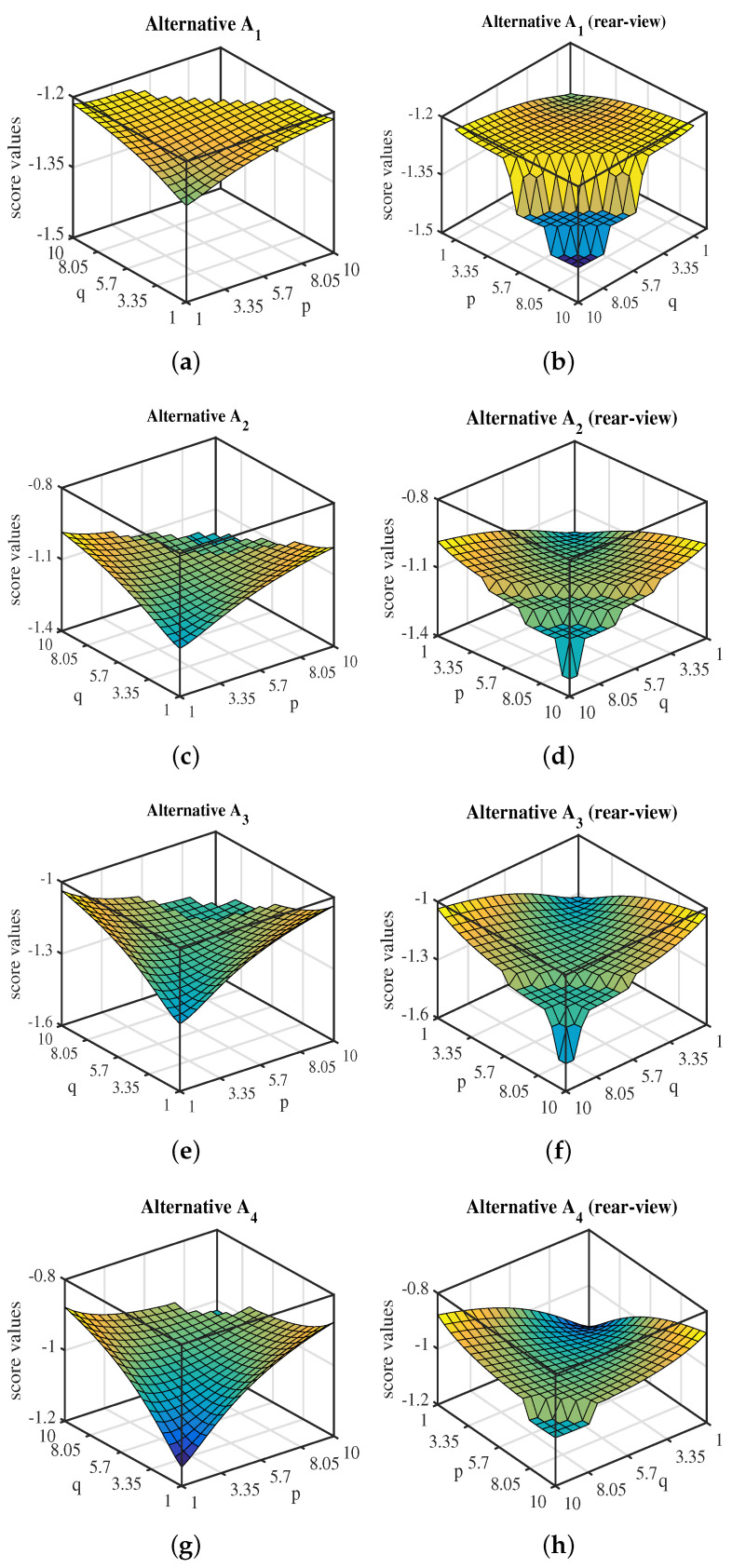
Score values of alternative for different values of *p* and *q*.

Figure 2 gives an outlook to influence of variable *p* and *q* values on the scores obtained by utilizing WCIFBM operator. It clearly shows that the score function possess different values for different values of parameters *p* and *q* ranging between 1 to 10. For an alternative $A_{1}$, the score value lies between −1.45 to −1.2 while from its corresponding rear-view plot, it is seen that the score value undergoes surface change thrice. The first surface starts at p=q=1 and ends at (the leftmost coordinate measure) p=10,q=4.5 having score value −1.226. The second surface starts at p=10 and q=5.5 having Sc=−1.353 whereas it ends at p=10,q=7.5 possessing Sc=−1.35. The third surface begins at p=10,q=8.5 bearing Sc=−1.412 and ends at p=10,q=10 with Sc=−1.41. The alternatives surface readings (Sr) with values of *p* and *q* are given in Table 5 along-with their corresponding score values. In this table Sr(d)(B) denotes the beginning of *d*th surface and Sr(d)(E) denotes ending of *d*th surface where “*d*” is an integer. It can be seen that $A_{4}$ has score values ranging over two surfaces whereas $A_{2}$ has score values spread over 4 surfaces with only one value on the 4th surface i.e., (10,10). On the other hand, both alternatives $A_{1}$ and $A_{3}$ covers three surfaces with $A_{3}$ having only one point i.e., (10,10) lying on 3rd surface.

### 5.3. Validity Test

To demonstrate our approach’s viability in the dynamic working environment, following test criteria, corroborated by Wang and Triantaphyllou [42], are accomplished

**Test criterion 1:** “If we replace the rating values of non-optimal alternative with worse alternative then the best alternative should not change, provided the relative weighted criteria remains unchanged.”
**Test criterion 2:** “Method should possess transitive nature.”
**Test criterion 3:** “When a given problem is decomposed into smaller ones and the same MADM method has been applied, then the combined ranking of the alternatives should be identical to the ranking of un-decomposed one.”

#### 5.3.1. Validity Check with Criterion 1

Since, ranking obtained from proposed approach is $A_{4}≻A_{2}≻A_{1}≻A_{3}$, then for testing the analogous nature of our approach under test criterion 1, the non-optimal alternative $A_{1}$ is replaced with a worst alternative $A_{1}^{\prime }$ where rating value of $A_{1}^{\prime }$ under the three considered criteria are expressed as {(〈[0.11,0.15], [0.60,0.70]〉, 〈0.30, 0.10〉); (〈[0.22,0.25], [0.45,0.55]〉, 〈0.40, 0.10〉) and (〈[0.20,0.28], [0.45,0.70]〉, 〈0.40, 0.15〉)}. Based on these observation, the proposed approach has been applied and hence the final score values of the alternatives are obtained as $\mathrm{Sc}(r_{1}^{\prime })=-1.4431$, $\mathrm{Sc}(r_{2})=-1.1989$, $\mathrm{Sc}(r_{3})=-1.3185$ and $\mathrm{Sc}(r_{4})=-1.1474$. Therefore, the ranking order is $A_{4}≻A_{2}≻A_{3}≻A_{1}^{\prime }$ in which the best alternative remains same as that of the proposed approach. Thus, our approach is fetching out consistent results with respect to the test criterion 1.

#### 5.3.2. Validity Check with Criteria 2 and 3

For checking validity corresponding to criteria 2 and 3, the fragmented MADM subproblems are taken as $\left\{A_{2},A_{3},A_{4}\right\}$, $\left\{A_{1},A_{3},A_{2}\right\}$ and $\left\{A_{2},A_{4},A_{1}\right\}$. Then, following the stated procedure of the approach their ranking is obtained as: $A_{4}≻A_{2}≻A_{3}$, $A_{2}≻A_{1}≻A_{3}$ and $A_{4}≻A_{2}≻A_{1}$, respectively. The overall ranking by clubbing all of them is $A_{4}≻A_{2}≻A_{1}≻A_{3}$ which is same as that of the results of the proposed original MADM problem, hence it beholds the transitive property. Therefore, the proposed method is valid under the test criterion 2 and test criterion 3.

### 5.4. Comparative Studies

In order to justify the superiority of our proposed mean operator with respect to the existing approaches namely, Bonferroni mean operator [33,36], averaging operator [11,24], geometric operator [19,23], ranking method [13,15,16], an analysis has been conducted under the IVIFSs environment by taking intuitionistic fuzzy judgements of CIFSs as zero and the weight vector is $\kappa ={\left(0.20,0.38,0.42\right)}^{T}$. The optimal score values and the ranking order of the alternatives are summarized in Table 6. From this table, we observed that the best alternative coincides with the proposed approach results which validate the stability of the approach with respect to state-of-art. Compared with these existing approaches with general intuitionistic sets (IVIFSs or IFSs), the proposed decision-making method under cubic intuitionistic fuzzy set environment contains much more evaluation information on the alternatives by considering both the IVIFSs and IFSs simultaneously, while the existing approaches contain either IFS or IVIFS information. Therefore, the approaches under the IVIFSs or IFSs may lose some useful information, either IVIFNs or IFNs, of alternatives which may affect the decision results. Furthermore, it is noted from the study that the computational procedure of the proposed approach is different from the existing approaches under the different environment, but the proposed result in this paper is more rational to reality in the decision process due to the consideration of the consistent priority degree between the pairs of the arguments. In the end, it is concluded that proposed operators consider the decision makers’ parameters *p* and *q*, which provide, the more choices to the decision makers to avail their desirable alternatives depending upon the different score values of the alternatives for the different parametric values of *p* and *q*. Also, the corresponding studies under the IVIFS or IFS environment can be considered as a special case of the proposed operators. Finally, the existing decision-making methods cannot deal with the decision-making problem with CIFS.

## 6. Conclusions

The cubic intuitionistic fuzzy set is an important tool for dealing with the uncertainty and fuzziness by expressing the interval-valued intuitionistic fuzzy number and intuitionistic fuzzy value simultaneously during the decision-making process. The aim of this manuscript is to present an aggregation operator named as Bonferroni mean whose remarkable characteristic is to capture the relationships between the individual arguments. For this, we have presented two BM operators i.e., the CIFBM operator and the WCIFBM operator, to aggregate the different preferences of experts over the different attributes under CIFS environment. Also, various desirable characteristics of these operators are studied. Finally, an approach for solving the decision-making problems has been presented by taking different values of parameters *p* and *q*, which makes the proposed operators more flexible and offers the various choices to the decision-maker for assessing the decisions. A comparative study with some existing operators shows that the proposed operators and their corresponding techniques provide a more stable, practical, and optimistic nature to the decision-maker during the aggregation process. Thus, we conclude that the proposed operators can be applied as an alternative way to solve the problem in real-life situations. In the future, we will extend the proposed approach to some other uncertain environment [43,44,45].

## Figures and Tables

**Table 1:** Rating values of the alternatives in terms of CIFNs.

Alternatives	$C_{1}$	$C_{2}$	$C_{3}$
$A_{1}$	$\left(\langle [0.50,0.60],[0.10,0.20]\rangle ,\langle 0.40,0.20\rangle \right)$	$\left(\langle [0.20,0.30],[0.40,0.50]\rangle ,\langle 0.30,0.20\rangle \right)$	$\left(\langle [0.40,0.60],[0.20,0.30]\rangle ,\langle 0.20,0.35\rangle \right)$
$A_{2}$	$\left(\langle [0.20,0.30],[0.40,0.50]\rangle ,\langle 0.40,0.60\rangle \right)$	$\left(\langle [0.15,0.25],[0.30,0.35]\rangle ,\langle 0.20,0.30\rangle \right)$	$\left(\langle [0.20,0.40],[0.10,0.20]\rangle ,\langle 0.40,0.50\rangle \right)$
$A_{3}$	$\left(\langle [0.50,0.60],[0.20,0.30]\rangle ,\langle 0.20,0.40\rangle \right)$	$\left(\langle [0.40,0.60],[0.25,0.35]\rangle ,\langle 0.30,0.40\rangle \right)$	$\left(\langle [0.50,0.70],[0.10,0.15]\rangle ,\langle 0.30,0.50\rangle \right)$
$A_{4}$	$\left(\langle [0.30,0.50],[0.10,0.30]\rangle ,\langle 0.10,0.30\rangle \right)$	$\left(\langle [0.40,0.55],[0.15,0.20]\rangle ,\langle 0.30,0.40\rangle \right)$	$\left(\langle [0.40,0.50],[0.20,0.30]\rangle ,\langle 0.45,0.35\rangle \right)$

**Table 2:** Normalized decision ratings.

Alternatives	$C_{1}$	$C_{2}$	$C_{3}$
$A_{1}$	$\left(\langle [0.10,0.20],[0.50,0.60]\rangle ,\langle 0.20,0.40\rangle \right)$	$\left(\langle [0.20,0.30],[0.40,0.50]\rangle ,\langle 0.30,0.20\rangle \right)$	$\left(\langle [0.20,0.30],[0.40,0.60]\rangle ,\langle 0.35,0.20\rangle \right)$
$A_{2}$	$\left(\langle [0.40,0.50],[0.20,0.30]\rangle ,\langle 0.60,0.40\rangle \right)$	$\left(\langle [0.15,0.25],[0.30,0.35]\rangle ,\langle 0.20,0.30\rangle \right)$	$\left(\langle [0.10,0.20],[0.20,0.40]\rangle ,\langle 0.50,0.40\rangle \right)$
$A_{3}$	$\left(\langle [0.20,0.30],[0.50,0.60]\rangle ,\langle 0.40,0.20\rangle \right)$	$\left(\langle [0.40,0.60],[0.25,0.35]\rangle ,\langle 0.30,0.40\rangle \right)$	$\left(\langle [0.10,0.15],[0.50,0.70]\rangle ,\langle 0.50,0.30\rangle \right)$
$A_{4}$	$\left(\langle [0.10,0.30],[0.30,0.50]\rangle ,\langle 0.30,0.10\rangle \right)$	$\left(\langle [0.40,0.55],[0.15,0.20]\rangle ,\langle 0.30,0.40\rangle \right)$	$\left(\langle [0.20,0.30],[0.40,0.50]\rangle ,\langle 0.35,0.45\rangle \right)$

**Table 3:** Effects on the ranking with the variation of the parameters *p* and *q*.

*p*	*q*	Sc$\left(A_{1}\right)$	Sc$\left(A_{2}\right)$	Sc$\left(A_{3}\right)$	Sc$\left(A_{4}\right)$	Ranking Order
p=1	q=1	−1.2942	−1.1989	−1.3185	−1.1474	$A_{4}≻A_{2}≻A_{1}≻A_{3}$
	q=2	−1.2815	−1.1726	−1.2825	−1.1047	$A_{4}≻A_{2}≻A_{1}≻A_{3}$
	q=3	−1.2674	−1.1386	−1.2335	−1.0582	$A_{4}≻A_{2}≻A_{3}≻A_{1}$
	q=4	−1.2552	−1.1065	−1.1881	−1.0172	$A_{4}≻A_{2}≻A_{3}≻A_{1}$
p=2	q=1	−1.2815	−1.1726	−1.2825	−1.1047	$A_{4}≻A_{2}≻A_{1}≻A_{3}$
	q=2	−1.2776	−1.1715	−1.2872	−1.0956	$A_{4}≻A_{2}≻A_{1}≻A_{3}$
	q=3	−1.2683	−1.1534	−1.2632	−1.0693	$A_{4}≻A_{2}≻A_{3}≻A_{1}$
	q=4	−1.2588	−1.1304	−1.2300	−1.0397	$A_{4}≻A_{2}≻A_{3}≻A_{1}$
p=3	q=1	−1.2674	−1.1386	−1.2335	−1.0582	$A_{4}≻A_{2}≻A_{3}≻A_{1}$
	q=2	−1.2683	−1.1534	−1.2632	−1.0693	$A_{4}≻A_{2}≻A_{3}≻A_{1}$
	q=3	−1.2628	−1.1487	−1.2627	−1.0594	$A_{4}≻A_{2}≻A_{3}≻A_{1}$
	q=4	−1.2559	−1.1357	−1.2456	−1.0417	$A_{4}≻A_{2}≻A_{3}≻A_{1}$
p=4	q=1	−1.2552	−1.1065	−1.1881	−1.0172	$A_{4}≻A_{2}≻A_{3}≻A_{1}$
	q=2	−1.2588	−1.1304	−1.2300	−1.0397	$A_{4}≻A_{2}≻A_{3}≻A_{1}$
	q=3	−1.2559	−1.1357	−1.2456	−1.0417	$A_{4}≻A_{2}≻A_{3}≻A_{1}$
	q=4	−1.2511	−1.1314	−1.2447	−1.0340	$A_{4}≻A_{2}≻A_{3}≻A_{1}$

**Table 4:** Ranking order of the alternatives with accuracy value at $q^{\prime }$.

Figure	Value of $q^{\prime }$	Accuracy Value at $q=q^{\prime }$	Ranking Order		
			When $q<q^{\prime }$	When $q=q^{\prime }$	When $q>q^{\prime }$
1a	2.0307	H$(A_{1})=1.6286$ , H$(A_{3})=1.6410$	$A_{4}≻A_{2}≻A_{1}≻A_{3}$	$A_{4}≻A_{2}≻A_{3}≻A_{1}$	$A_{4}≻A_{2}≻A_{3}≻A_{1}$
1b	2.742	H$(A_{1})=1.6269$ , H$(A_{3})=1.7417$	$A_{4}≻A_{2}≻A_{1}≻A_{3}$	$A_{4}≻A_{2}≻A_{3}≻A_{1}$	$A_{4}≻A_{2}≻A_{3}≻A_{1}$
1c	-	-		$A_{4}≻A_{2}≻A_{3}≻A_{1}$	
1d	-	-		$A_{4}≻A_{2}≻A_{3}≻A_{1}$	

**Table 5:** Surface readings (Sr’s) for each alternative’s rear view.

	$A_{1}$		$A_{2}$		$A_{3}$		$A_{4}$	
	(p,q)	Sc$\left(A_{1}\right)$	(p,q)	Sc$\left(A_{2}\right)$	(p,q)	Sc$\left(A_{3}\right)$	(p,q)	Sc$\left(A_{4}\right)$
Sr(1)(B)	(1,1)	−1.294	(1,1)	−1.199	(1,1)	−1.302	(1,1)	−1.147
Sr(1)(E)	(10,4.5)	−1.226	(10,4)	−1.053	(10,5.5)	−1.166	(10,7.5)	−0.9665
Sr(2)(B)	(10,5.5)	−1.353	(10,5)	−1.102	(10,6.5)	−1.227	(10,8)	−1.029
Sr(2)(E)	(10,7.5)	−1.35	(10,6.5)	−1.115	(10,8.5)	−1.239	(10,10)	−1.027
Sr(3)(B)	(10,8.5)	−1.412	(10,7.5)	−1.173	(10,10)	−1.458	–	–
Sr(3)(E)	(10,10)	−1.41	(10,9)	−1.174	(10,10)	−1.458	–	–
Sr(4)(B)	–	–	(10,10)	-1.323	–	–	–	–
Sr(4)(E)	–	–	(10,10)	-1.323	–	–	–	–

**Table 6:** Comparison analysis with some of the existing approaches.

Comparison with	Score Values				Ranking
	$A_{1}$	$A_{2}$	$A_{3}$	$A_{4}$	
Xu and Chen [33]	−0.7152	−0.5847	−0.6880	−0.5830	$A_{4}≻A_{2}≻A_{3}≻A_{1}$
Shi and He [36]	−0.2680	−0.0685	−0.2244	−0.0301	$A_{4}≻A_{2}≻A_{3}≻A_{1}$
Wang and Liu [11]	−0.2593	−0.0635	−0.1552	0.0194	$A_{4}≻A_{2}≻A_{3}≻A_{1}$
Chen et al. [24]	−0.2633	−0.0630	−0.1425	0.0096	$A_{4}≻A_{2}≻A_{3}≻A_{1}$
Chen et al. [23]	−0.2608	−0.0613	−0.2154	−0.0315	$A_{4}≻A_{2}≻A_{3}≻A_{1}$
Sivaraman et al. [15]	−0.2120	−0.0792	−0.1910	0.0214	$A_{4}≻A_{2}≻A_{3}≻A_{1}$
Wan et al. [19]	−0.2610	−0.0705	−0.1835	−0.0100	$A_{4}≻A_{2}≻A_{3}≻A_{1}$
Dugenci [16]	0.7940	0.7316	0.7693	0.7106	$A_{4}≻A_{2}≻A_{3}≻A_{1}$
Garg [13]	0.1082	0.1101	0.1230	0.1649	$A_{4}≻A_{3}≻A_{2}≻A_{1}$
